# Hypoxia-induced gene expression results from selective mRNA partitioning to the endoplasmic reticulum

**DOI:** 10.1093/nar/gkv167

**Published:** 2015-03-08

**Authors:** Jonas J. Staudacher, Isabel S. Naarmann-de Vries, Stefanie J. Ujvari, Bertram Klinger, Mumtaz Kasim, Edgar Benko, Antje Ostareck-Lederer, Dirk H. Ostareck, Anja Bondke Persson, Stephan Lorenzen, Jochen C. Meier, Nils Blüthgen, Pontus B. Persson, Alexandra Henrion-Caude, Ralf Mrowka, Michael Fähling

**Affiliations:** 1 Charité - Universitätsmedizin Berlin, Institut für Vegetative Physiologie, Charitéplatz 1, D-10117 Berlin, Germany; 2 University Hospital Aachen, RWTH Aachen University, Department of Intensive and Intermediate Care, Experimental Research Unit, D-52074 Aachen, Germany; 3 Humboldt Universität zu Berlin, Institut für Theoretische Biologie, D-10115 Berlin, Germany; 4 Charité - Universitätsmedizin Berlin, Institut für Pathologie, D-10117 Berlin, Germany; 5 Universitätsklinikum Jena, Klinik für Innere Medizin III, AG Experimentelle Nephrologie, D-07743 Jena, Germany; 6 Max Delbrück Center for Molecular Medicine, RNA Editing and Hyperexcitability Disorders Helmholtz Group, D-13125 Berlin, Germany; 7 TU Braunschweig, Zoological Institute, Division of Cell Physiology, D-38106 Braunschweig, Germany; 8 Hôpital Necker-Enfants Malades, Université Paris Descartes, Institut National de la Santé et de la Recherche Médicale (INSERM) UMR1163 and Imagine Foundation, 75015 Paris, France

## Abstract

Protein synthesis is a primary energy-consuming process in the cell. Therefore, under hypoxic conditions, rapid inhibition of global mRNA translation represents a major protective strategy to maintain energy metabolism. How some mRNAs, especially those that encode crucial survival factors, continue to be efficiently translated in hypoxia is not completely understood. By comparing specific transcript levels in ribonucleoprotein complexes, cytoplasmic polysomes and endoplasmic reticulum (ER)-bound ribosomes, we show that the synthesis of proteins encoded by hypoxia marker genes is favoured at the ER in hypoxia. Gene expression profiling revealed that transcripts particularly increased by the HIF-1 transcription factor network show hypoxia-induced enrichment at the ER. We found that mRNAs favourably translated at the ER have higher conservation scores for both the 5′- and 3′-untranslated regions (UTRs) and contain less upstream initiation codons (uAUGs), indicating the significance of these sequence elements for sustained mRNA translation under hypoxic conditions. Furthermore, we found enrichment of specific *cis*-elements in mRNA 5′- as well as 3′-UTRs that mediate transcript localization to the ER in hypoxia. We conclude that transcriptome partitioning between the cytoplasm and the ER permits selective mRNA translation under conditions of energy shortage.

## INTRODUCTION

When inadequate oxygen supply lowers relative tissue oxygen tensions, the hypoxia inducible factor (HIF), a heterodimeric transcription factor activating glycolysis and angiogenesis, plays a pivotal role in the adaptation of gene expression (1–8). Nevertheless, energy shortage represses cellular metabolism including mRNA translation (9,10). In hypoxia, protein synthesis rates drop to 5–40% (11) and thus become a bottleneck for gene expression, which represents a major protective strategy to maintain energy metabolism (10,12). mRNA translation is among the most energy-consuming processes in the cell and the majority of translational regulation occurs at the level of initiation, which is thought to be the rate-limiting step (13,14). It has been shown that protein synthesis has a significant impact on hypoxia tolerance, supporting the pivotal role of mRNA translation in cellular adaptation to hypoxia (15,16). Global suppression of protein synthesis occurs through phosphorylation of the α-subunit of eukaryotic initiation factor 2 (eIF2-α) and inhibition of mammalian target of rapamycin (mTOR) under prolonged hypoxia, both leading to repression of 5′-cap-dependent initiation of mRNA translation (17–19). Notably, despite a global repression of gene expression, several proteins, many of which are HIF targets, show increased expression.

Some *trans*-acting factors such as RNA-binding proteins (RBPs) and miRNAs that modulate hypoxic gene expression at the post-transcriptional level have been described for individual mRNAs (20,21). To date, only few alternative mechanisms that initiate mRNA translation under global translation suppression have been described. These include internal ribosome entry segments (IRESs), upstream open reading frames (uORFs) and in addition to the poly-A tail length, a number of unique elements in the 3′ UTR (22).

5′-cap-independent initiation of mRNA translation mediated by IRESs is an attractive concept (23,24). IRESs interact with IRES-*trans*-acting factors, which remodel IRESs into permissive 40S ribosomal subunit binding structures (22). While this mechanism surely plays a role in regulating mRNA translation in hypoxia, it cannot explain the whole regulation observed as only a subset of hypoxia responsive mRNAs carry an IRES. Indeed, increased mRNA translation has been demonstrated for several mRNAs lacking an IRES during hypoxia (25–27). Thus, IRES-independent mechanisms have been suggested for sustained protein synthesis during hypoxia. For instance, the HIF-2α–RBM4–eIF4E2 complex captures the mRNA 5′-cap and targets transcripts to polysomes for active translation in hypoxic glioblastoma cells (28). A 5′-cap-dependent selective translation in hypoxia has also been linked to HIF-1 mediated upregulation of eIF4E1 in breast cancer cells (29). Moreover, a HILDA (hypoxia-inducible hnRNP-L–DRBP76–hnRNP-A2/B1) complex has been shown to coordinate a three-element RNA switch, enabling VEGFA mRNA translation in hypoxia (30).

Another central mechanism of translational control is represented by uORFs, mRNA elements defined by an AUG start codon in the 5′-untranslated region (UTRs) that is out-of-frame (uAUG). uORFs have been proposed as regulatory elements in the 5′UTR of specific mRNAs that convey translational repression, which can be bypassed at reduced eIF2-GTP when eIF2α phosphorylation is enhanced under nutrition- or ER-stress (31). Although uAUGs do not necessarily represent regulatory uORFs, uAUGs *per se* have a substantial impact on protein expression (32). In humans and rodents, approximately half of the transcripts contain uAUGs and their presence correlates with reduced protein expression (33). A systematic analysis of single-nucleotide mutations in yeast revealed that major changes in gene expression were not due to mutations in transcription factor binding sites, but newly generated uAUGs (32). However, whether translational repression or activation via uAUGs contributes to the regulation of mRNA translation in hypoxia is an open issue.

An alternative option for transcript-specific protein synthesis is the spatial organization of mRNA translation between the cytoplasm and the endoplasmic reticulum (ER), which serves as a regulatory mechanism during stress (34). The ER is a dynamic compartment, which facilitates translation of a global mRNA population, partially independent of the signal-recognition particle pathway (35–38). Under stress conditions, spatial redistribution of translational activity between the cytoplasm and the ER results from selective inhibition of cytoplasmic mRNA translation, while translation continues at the ER (34,35). Thus, mRNA partitioning between the cytoplasm and the ER has been proposed as a candidate mechanism for translation of specific mRNAs during hypoxia (39). Currently, the mechanisms of protein synthesis during hypoxia have been studied uniquely focusing on the cytoplasmic compartment.

To shed light on how and where mRNA translation takes place in hypoxia, we analysed RNA partitioning between the cytoplasm and the ER. We demonstrate that the ER plays a crucial role in the adaptation of gene expression during hypoxia, especially for factors belonging to the gene ontologies (GOs) ‘hypoxia response’, ‘glycolysis’ or ‘HIF-1α transcription factor network’. We further show that transcripts that are favourably translated at the ER during hypoxia, especially HIF-targets, contain less uAUGs in their 5′-UTRs, while both their mRNA 5′- and 3′UTRs show higher conservation scores. Subsequent *cis*-element enrichment analysis revealed that both, the 5′- and the 3′-UTRs, carry specific motifs that mediate ER localization and, thus, sustained translation efficiency in hypoxia.

## MATERIALS AND METHODS

### Cell culture

Human fibrosarcoma HT1080 cells from the DSMZ, Germany (ACC 315) were cultured under control (21% O_2_, 5% CO_2_, 37°C) or hypoxic (1% O_2_, 5% CO_2_, 37°C) conditions as described (27).

### RNA and protein isolation

Total RNA was isolated using RNA-Bee™ (CS-105B; AMS Biotechnology, Europe) according to the manufacturer's protocol. Total cellular protein extracts were prepared and analysed by western blotting as described elsewhere (40). The following primary antibodies were used: P4HB/PDI (Acris, #AP17615PU-N); P4H-α(I) (Acris, #AF0210); β-Actin (Millipore, #MAB1501R); HIF-1α (BD Biosciences, #610959); BLID (Abnova, #H00414899-B01); Tubulin β (Proteintech™, #10063–2-AP); GAPDH (Acris, #BM439); Aromatase/Cytochrome P450 (Acris, #AP00001PU-N). Secondary antibodies were used according to the manufacturers’ recommendations.

### Fractional RNA/protein isolation

For RNA isolation from subcellular fractions (e.g. polysomal gradient centrifugation, ER isolation) cells were pre-treated with cycloheximide (100 μg/ml) for 10 min. For RNA and protein isolation cells were quickly washed with ice-cold phosphate buffered saline. Western blotting was performed as described elsewhere (27). Total RNA was prepared using RNA-Bee (Biozol Diagnostica Vertrieb GmbH) according to the manufacturer's protocol.

Cytosolic extracts conforming to 10 000 x *g* supernatants (S10) were prepared using a lysis buffer (20 mM Tris, pH 7.4, 150 mM KCl, 30 mM MgCl_2_, 0.25% Nonidet P40, 20 μg/ml cycloheximide, 200 U/ml RNaseOUT [Invitrogen], 1 mM Dithiothreitol (DTT), 1× complete protease-inhibitor-mix [Roche Diagnostics]). After 2 min incubation on ice, cells were centrifuged at 10 000 x *g*, 4°C for 10 min (S10).

### Sucrose density gradient polysome analysis

Cytoplasmic extracts (S10) were layered onto 12 ml of a linear 0.5–1.5 M (17–51%) sucrose gradient and centrifuged (2 h, 36 000 rpm Beckman SW-41). The ribosomal profile was determined at 254 nm (A_254_) for each evaluation. Sucrose gradients were split into 12 subfractions. RNA was isolated using the E.Z.N.A. RNA Total Kit (#OMEGR6834-02, VWR International) according to the manufacturer's protocol. An external standard (a synthetic *in vitro* transcript) was added prior to RNA isolation as a technical control. For isolation of pooled polysomal and non-polysomal fractions, cytoplasmic (S10) extracts were ultra-centrifuged for 1.5 h at 4°C and 100 000 x *g*. Sediments represent a polysomal and supernatants represent a non-polysomal fraction.

### ER-RNA isolation

Isolation of an ER fraction was performed using the ER0100 Sigma Endoplasmic Reticulum Isolation Kit (Sigma-Aldrich). RNA was extracted from the ER fraction using RNA-Bee™ following the manufacturer's instructions.

### RNA quantification by real-time PCR (qPCR)

First-strand cDNA synthesis was performed with 1 μg of total RNA using random primers and Superscript II reverse transcriptase (Invitrogen). Quantitative real-time PCR (qPCR) analysis was performed in triplicate with the GeneAmp 5700 system (Applied Biosystems) according to the manufacturer's instructions (for primer sequences, see Supplemental Materials & Methods section). mRNA expression levels were normalized to 18S rRNA using the ΔC_t_ method.

### Immunofluorescence staining and fluorescence *in situ* hybridization (IF-FISH)

FISH combined with immunofluorescence staining was performed as described using FISH probes for VEGF and β-Actin mRNAs (41). FISH probes for HIF-1α, P4HA1, BLID and Luciferase mRNAs were: HIF-1α: ATGTGGAAGTGGCAACTGATGAGCAAGCTC, P4HA1: TGTCCCATTCATCCTCCTGTTTAGCTGGAG, BLID: CTATCCATCCTGTGTAGAGCACACACTCAG, Luciferase: GGAGGTAGATGAGATGTGACGAACGTGTAC. Antibodies were purchased from Abcam (GM130: ab52649, Calnexin: ab22595) and Santa Cruz (rpL19: sc-100830). Microscopy was performed on an Apotome 2 (Zeiss), images were acquired with AxioVision (Zeiss) and intensity profiles with ImageJ.

### Microarrays and GO enrichment analysis

Pooled total-RNA and ER-RNA from five independent experiments under control or hypoxic conditions were used for the microarray experiments. For global gene expression profile analysis, the Affymetrix human Gene 1.0 ST (Affymetrix Inc., Santa Clara, CA, USA) was used according to the manufacturer's recommendations. Arrays were processed at the genome analysis facility of the Charité (Charité LGFC). After normalization by RMA, genes below the 50% expression quantile were disregarded. A significant change in expression was defined by: (i) Fold change (FC) > 1.4 to non-hypoxic control and (ii) *z*-score > 3. The mean-standard deviation loess model for the *z*-score estimation was determined for treatment and control groups separately as described elsewhere (42). GO enrichment analysis was performed using the WEB-based GEne SeT AnaLysis Toolkit (WEBGESTALT; http://bioinfo.vanderbilt.edu/webgestalt/analysis.php) (43). HuGene 1.0 ST Probe IDs of significantly regulated candidates in specific groups according to expression and localization were used for GO enrichment analysis using the following parameters: Organism: hsapiens, Id Type: affy_hugene_1_0_st_v1, Ref Set: affy_hugene_1_0_st_v1, Statistic: Hypergeometric, Significance Level: Top10, MTC: BH, Minimum: 2.

Microarray data were submitted to the Gene Expression Omnibus [identifier GSE49029].

### Motif enrichment analysis in subgroups

To identify potential short regulatory motifs in the UTRs of the subgroups we used the DREME tool (44) of the MEME suite (45) version 4.8.1., which was downloaded from the meme home page [http://meme.nbcr.net/meme/] and installed locally on the UNIX operating system. Briefly, DREME (Discriminative Regular Expression Motif Elicitation) finds short motifs that are enriched in a set of DNA sequences in comparison to a reference set (44). The statistics of the DREME program relies on the Fisher Exact Test. We used the complete human 5′-UTR or the complete 3′-UTR set as reference sets for the specific 3′- or 5′-UTR subgroups, respectively.

### Reporter gene assays

For reporter gene assays, the 5′- or 3′-UTRs of luciferase mRNA (pGL3-promotor vector, Promega, constitutive SV40 promoter) were modified. Enriched motifs were inserted as 32–40 oligonucleotides carrying the motif in their centers. pGL3-promoter luciferase 5′- and 3′-UTRs with inserted native or mutated motifs were derived from gene synthesis, cloned and verified by sequencing. The original pGL3-promoter served as a control. Gene synthesis, cloning and sequencing were performed by ATG:biosynthetics GmbH (D-79249 Merzhausen Germany). Reporter gene assays using modified firefly luciferase constructs normalized to renilla luciferase (phRL-TK vector) were performed as described (27).

### Transfection of HT1080 cells for IF-FISH analysis

HT1080 cells (1 × 10^6^ cells in Dulbecco's modified Eagle's medium without fetal bovine serum (FBS) and antibiotics) were electroporated at 250 μF and 400 V (Gene Pulser Xcell Electroporation system, Bio-Rad) with 10 μg DNA (pGL3p, pGL3p-5′UTR-cis1_wt_, pGL3p-5′UTR-cis1_mut_, pGL3p-3′UTR-cis1_wt_, pGL3p-3′UTR-cis1_mut_, pGL3p-3′UTR-cis22_wt_, pGL3p-3′UTR-cis22_mut_) or mock transfected. 1 × 10^5^ cells were seeded per well onto a 24-well plate. At 8 h post-transfection, hypoxia treatment was started; at 44 h post-transfection cells were subjected to IF-FISH.

### Estimation of UTR length and UTR specific conservation score

We obtained the sequences of all human 5′- and 3′-UTRs of each transcript with the Biomart tool (46) of the ENSEMBL project (www.ensembl.org). Length was calculated for each UTR of each transcript using an in-house developed software tool. We only considered UTRs with a length of at least 30 nt for further analysis. For calculation of average evolutionary conservation score, we first obtained the basewise conservation score for the complete human genome from USCS (http://hgdownload.soe.ucsc.edu/goldenPath/hg19/phyloP46way/) that is based on algorithms that use multiple species comparisons (47,48). We then mapped the genome- and basewise conservation score onto the 3′-UTR and 5′-UTR positions of each transcript with a memory-optimized in-house software tool based on the c++ programming language using the c++ standard template library.

Boxplots were generated and statistical analysis was performed using the open source statistical package R (version 2.15.3) obtained from (http://www.r-project.org) with the Rcmdr package (http://socserv.mcmaster.ca/jfox/Misc/Rcmdr/). Statistical analysis is based on a linear ANOVA model. *Post hoc* analysis was performed with the ‘Tukey Contrasts—Multiple Comparisons of Means’ and the adjusted *P*-values are reported for the *post hoc* multiple comparisons.

### AUG score

The AUG number and UTR length were determined from all 5′-UTR transcripts. The AUG score represents the ratio of the number of uAUG to the length of the respective UTR. For each human transcript we calculated the AUG score with an in-house developed software tool. The gene-specific AUG score was analysed with a non-parametric two factor ANOVA (Scheirer-Ray-Hare-Test) with respect to location and expression level with the R statistical software tool (http://www.r-project.org/).

### Statistics

If not indicated otherwise, values are presented as means ± S.D. Students’ paired *t*-test was applied and *P*-values <0.05 were considered significant.

## RESULTS

### The expression level of hypoxia-induced proteins does not correlate with the association of their mRNAs to cytoplasmic ribosomes

**Figure 1.**
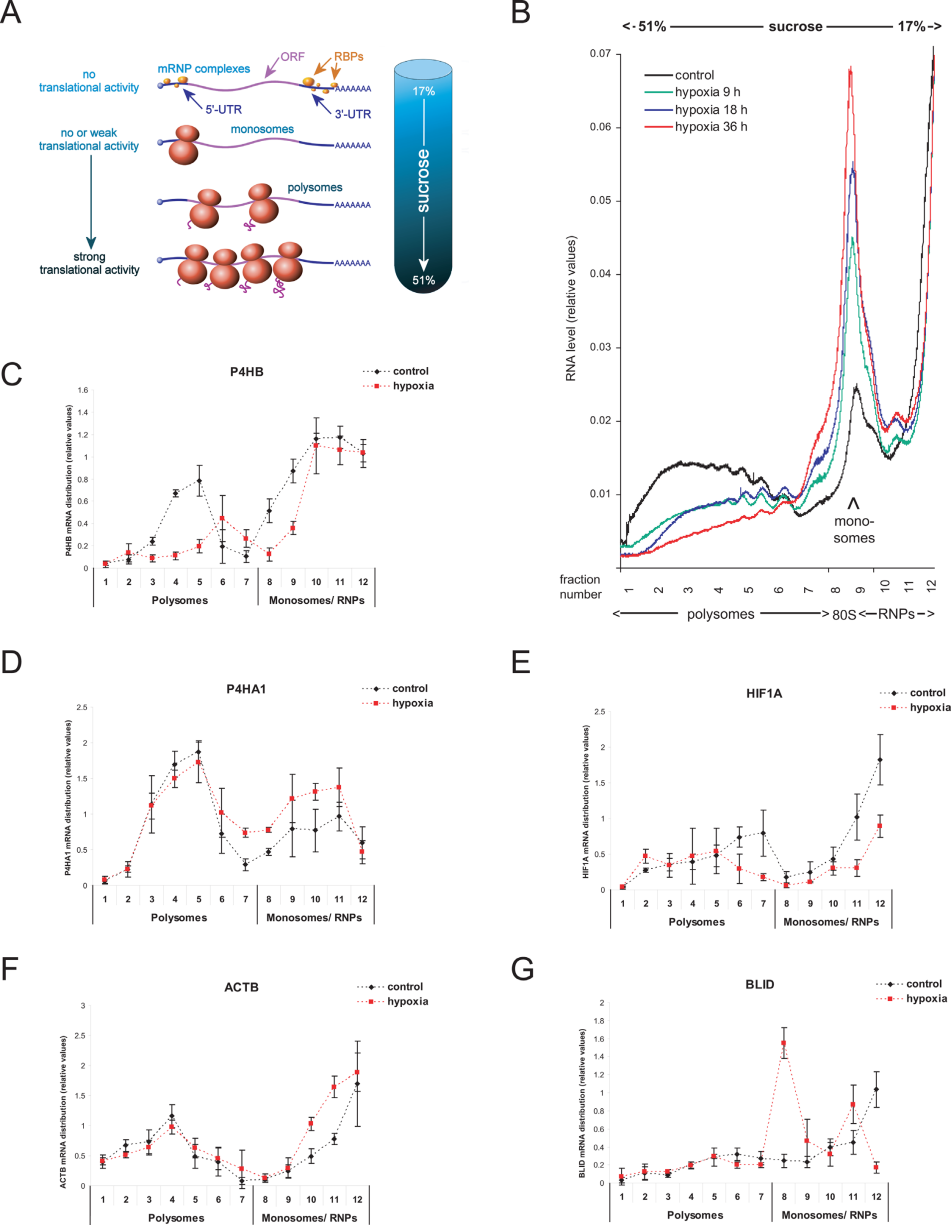
Polysomal gradient analysis. HT1080 cells were incubated under control (21% oxygen) or hypoxic (1% oxygen) conditions for up to 36 h. (**A**) Schematic overview of polysomal gradient analysis, adopted from (39). (**B**) Typical ribosomal profiles after sucrose gradient ultra-centrifugation monitored at 254 nm absorbance from bottom (51% sucrose) to top (17% sucrose). (**C**–**G**) Quantification of candidate transcripts in extracts of cells grown under normoxia or 36 h of hypoxia (1 % oxygen) following fractionation of sucrose gradients as indicated in (1B). *n* = 3.

To determine the effects of hypoxia on global mRNA translation in human fibrosarcoma cells (HT1080), we examined polysomal profiles at various time points. As depicted in Figure 1A, fractionation of the different RNA–ribosome complexes was established based on their molecular weight using a sucrose density gradient to account for: (i) the translationally-active polysomes and (ii) the translationally-inactive monosomes and RNPs. The number of ribosomes per transcript reflects the protein synthesis rate per time unit and mRNA copy.

Analysis of the polysomal profiles for up to 36 h of hypoxia (1% oxygen) showed a continuous disaggregation of cytoplasmic polysomes as revealed by a prominent increase in the 80S monosomes as well as 60S and 40S ribosomal subunits (Figure 1B), consistently with the inhibition of global mRNA translation (10,49,50). Most prominent polysomal disassembly occurred at 36 h, which was thus selected as the time point for further analysis. The gradient was separated into 12 subfractions to dissect the distribution of specific mRNAs that are known to be either hypoxia-inducible or non-inducible. We reckoned that the increase in cellular protein content could either be due to: (i) elevated levels of mRNA bound to polysomes and/or (ii) to an increased number of ribosomes per transcript, as assessed by a shift to higher molecular-weight fractions.

The prolyl hydroxylase-β subunit (P4HB) exemplifies a transcript that undergoes typical ribosomal disassembling (Figure 1C). In contrast, prolyl 4-hydroxylase α subunit (P4HA1), which is known to be increasingly translated in hypoxia (27), displayed a broad distribution across the gradient with no notable increase in its mRNA level in the translationally-active polysomal fractions but rather an increase in the translationally-inactive fractions (Figure 1D). While P4HA1 mRNA showed a stable association with ribosomes under conditions of polysomal disaggregation, both the mRNA amount in polysomes and the number of ribosomes associated with the transcript remained unchanged. This observation contrasts with the fact that a change in at least one condition is necessary to yield an increase in the protein levels. This prompted us to conclude that increased protein synthesis does not solely occur in the cytoplasmic compartment, in contrast to previous report.

While hypoxic induction of HIF-1α is primarily due to enhanced protein stability (51), active translation of its mRNA has been assumed. Nevertheless, we observed a decrease in HIF-1α mRNA within the polysomal fractions following hypoxia, consistent with previous observations (52,53) (Figure 1E). Notably, HIF-1α mRNA is decreased in the polysomal as well as the non-polysomal fractions, indicating a decreased transcript level in the cytoplasmic compartment, in contrast to earlier observations showing that the overall HIF-1α mRNA level is unchanged during hypoxia.

Of the non-hypoxia-inducible transcripts tested, β-Actin (ACTB) and BH3-like motif containing cell death inducer (BLID) were unchanged in cytoplasmic polysomes (Figure 1F and G). Interestingly, this observation contrasts with the general polysomal disassembling shown by ribosomal profiles (Figure 1B) or distribution of specific mRNAs such as P4HB (Figure 1C). In fact, ACTB and BLID mRNAs contain several upstream translation initiation codons (uAUGs) in their 5′-UTRs that might lead to an overestimation of ribosomal assembling as an indicator of protein synthesis rates. However, BLID mRNA was especially increased in the cytoplasmic monosomal fraction only (Figure 1G). In fact, monosomes may result from the recruitment of ribosomal subunits to the transcript while translation initiation is being inhibited.

As a result, hypoxia-inducible candidates did not show an mRNA enrichment at polysomes neither an increase of ribosomal assembling. Thus, polysomal profiles from cytoplasmic extracts may not completely reflect alterations in mRNA translation following sustained hypoxia (see also Supplementary Figure S1). Therefore, the specific regulation of mRNA translation during hypoxia was established by comparing the overall mRNA expression level and localization of the transcripts. For polysomal gradient analysis, cytoplasmic extracts (supernatant fraction following 10 000 x *g* centrifugation; S10) were used. We, thus, also measured candidate mRNA levels in the sediment fraction obtained after S10 centrifugation by qPCR under control and hypoxic conditions. Strikingly, only the hypoxia-inducible candidates (i.e. HIF1A, P4HA1 and HK2) exhibited an increase in the transcript levels of the ER-containing fraction (Supplementary Figure S2). We, thus, conclude that under hypoxia, mRNAs can (i) be translationally inactive and form ribonucleoprotein (mRNP) complexes, (ii) associate with cytoplasmic ribosomes or (iii) interact with ribosomes at the ER.

### Selective association of hypoxia-induced transcripts with ER-bound ribosomes

**Figure 2.**
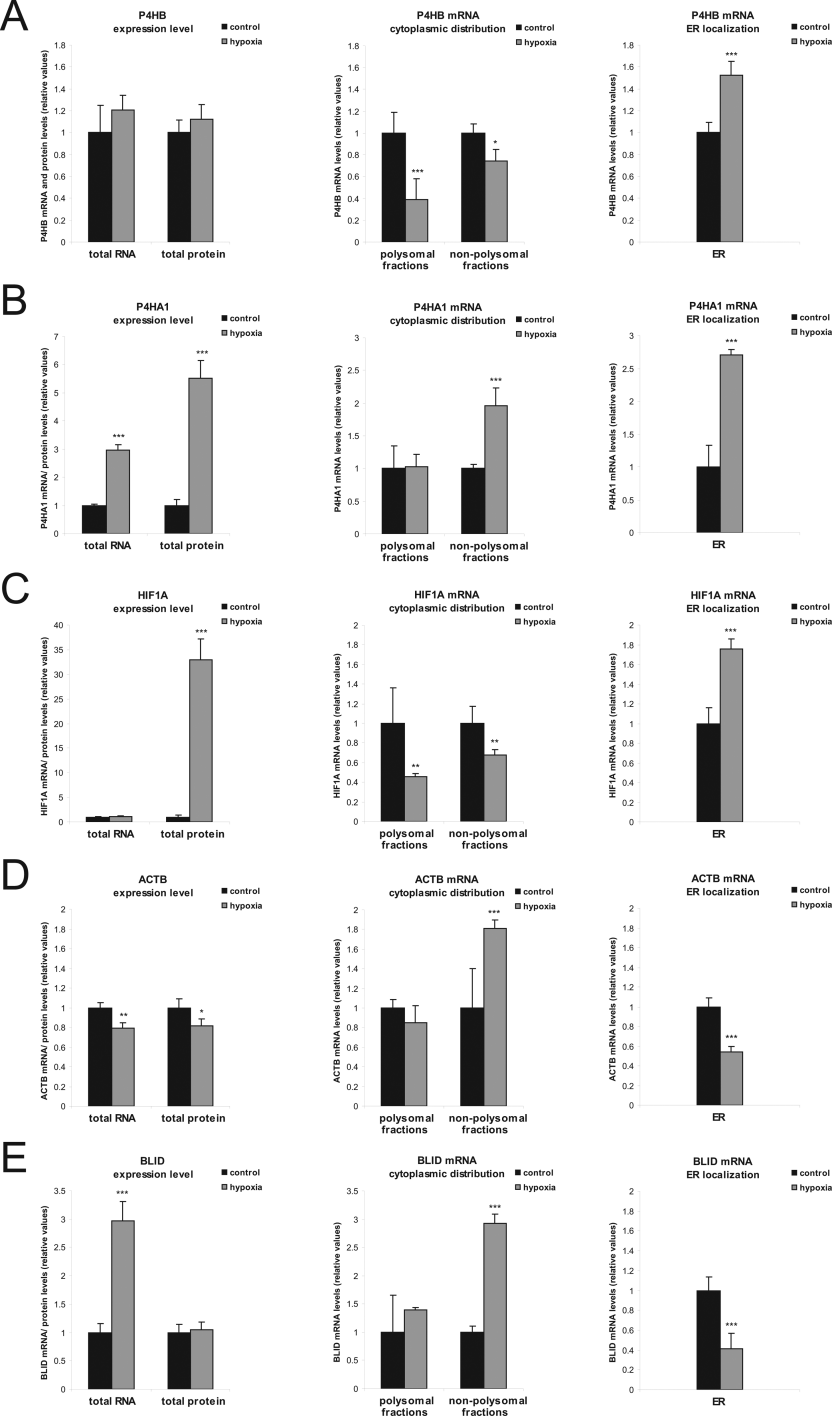
Estimation of gene expression rate and mRNA distribution in hypoxia. HT1080 cells were incubated under control and hypoxic conditions for 36 h. Gene expression levels (total RNA & total protein, left panel), mRNA localization in the cytoplasm (polysomal versus non-polysomal fractions, middle panel) and at the ER (right panel) are shown. (**A**–**E**) Total mRNA levels were determined by qPCR. Corresponding protein levels were estimated by western blotting as shown in Supplementary Figure S4. Levels for mRNAs in polysomal and non-polysomal fractions were determined following ultra-centrifugation of cytoplasmic extracts at 100 000 x *g*. ER-RNA was isolated using a commercially available ER-isolation kit. *n* = 5. *−*P* < 0.05, **−*P* < 0.01, ***−*P* < 0.001.

To assess the impact of mRNA partitioning on protein synthesis, we measured gene expression levels as determined by total RNA and protein levels as well as transcript levels of selected candidates in different subcellular compartments relevant for mRNA translation, i.e. cytoplasmic ribosomes or ER associated ribosomes. The cytoplasm was separated into translationally active sediment (polysomes) and translationally inactive supernatant (monosomes and RNP) fractions by ultra-centrifugation at 100 000 x *g*. A pure ER fraction was obtained by biochemical purification using a commercially available ER-isolation kit. Purity of the ER fraction was ensured by assessing the localization of cytoplasmic markers, i.e. GAPDH and β-Actin, and ER-specific markers i.e. aromatase and protein disulfide isomerase (PDI) (Supplementary Figure S3). Thus, cytoplasmic fractions generated by ultracentrifugation and biochemically purified ER were used for further analysis. A summary of the data is presented in Figure 2. Original western blotting results are presented in Supplementary Figure S4.

P4HB gene expression remained stable in hypoxia as indicated by unchanged total mRNA as well as total protein levels. Although the level of P4HB mRNA was decreased at cytoplasmic polysomes, it was increased in the ER fraction, suggesting a compensatory effect by translocation of P4HB mRNA to the ER (Figure 2A).

Following hypoxia, P4HA1 was increased at both the mRNA and the protein levels (Figure 2B). P4HA1 mRNA displayed stable association to cytoplasmic ribosomes under conditions of cytoplasmic polysomal disaggregation (Figure 2B). However, the amount of its mRNA in polysomes as well as the number of ribosomes associated with its transcript remained both unchanged (see: Figure 1D). Therefore, polysomal gradient analysis alone could not reflect the net effect seen by total RNA and protein quantification. An elevated protein level rather reflects the increased presence of the P4HA1 transcript at the ER.

Similarly, HIF-1α mRNA level was increased in the ER fraction, indicating a preference for ER localization under low oxygen tension. This is supported by a decrease of HIF-1α mRNA level in both polysomal and non-polysomal fractions with no change in its total mRNA level (Figure 2C). Thus, the decreased HIF-1α mRNA level observed by polysomal gradient analysis (see: Figure 1E) is explained by the partition of the transcript into the ER fraction. Together, HIF1A and P4HA1 mRNAs, which protein levels are markedly increased during hypoxia, are elevated at the ER following hypoxia. Therefore, the ER appears as the active compartment of protein synthesis in hypoxia.

In contrast, β-Actin mRNA is decreased in the ER fraction, which is in line with its increase in the cytoplasmic RNP fraction. These findings reflect an increase of untranslated β-Actin mRNA that is consistent with decreased β-Actin protein (Figure 2D). However, the decrease in β-Actin protein level is rather weak, which might be a result of stable β-Actin mRNA association in cytoplasmic polysomes.

Of note, albeit total BLID mRNA level increased three-fold in hypoxia (Figure 2E), which is comparable to the increase seen in P4HA1 mRNA (Figure 2B), the BLID mRNA increase was only detectable in the translationally inactive monosome/RNP fractions (compare Figure 2B and E, left and middle panel). At the ER, BLID mRNA was decreased, while its association to cytoplasmic ribosomes remained unchanged, consistent with unchanged BLID protein level (Figure 2E).

Altogether, these data are consistent with those obtained from cytoplasmic polysomal profiling. Hypoxia inducible candidates (HIF-1α, P4HA1) showed increased transcript levels in the ER fraction, while candidates that were not elevated at the protein level (β-Actin, BLID) were decreased at the ER.

**Figure 3.**
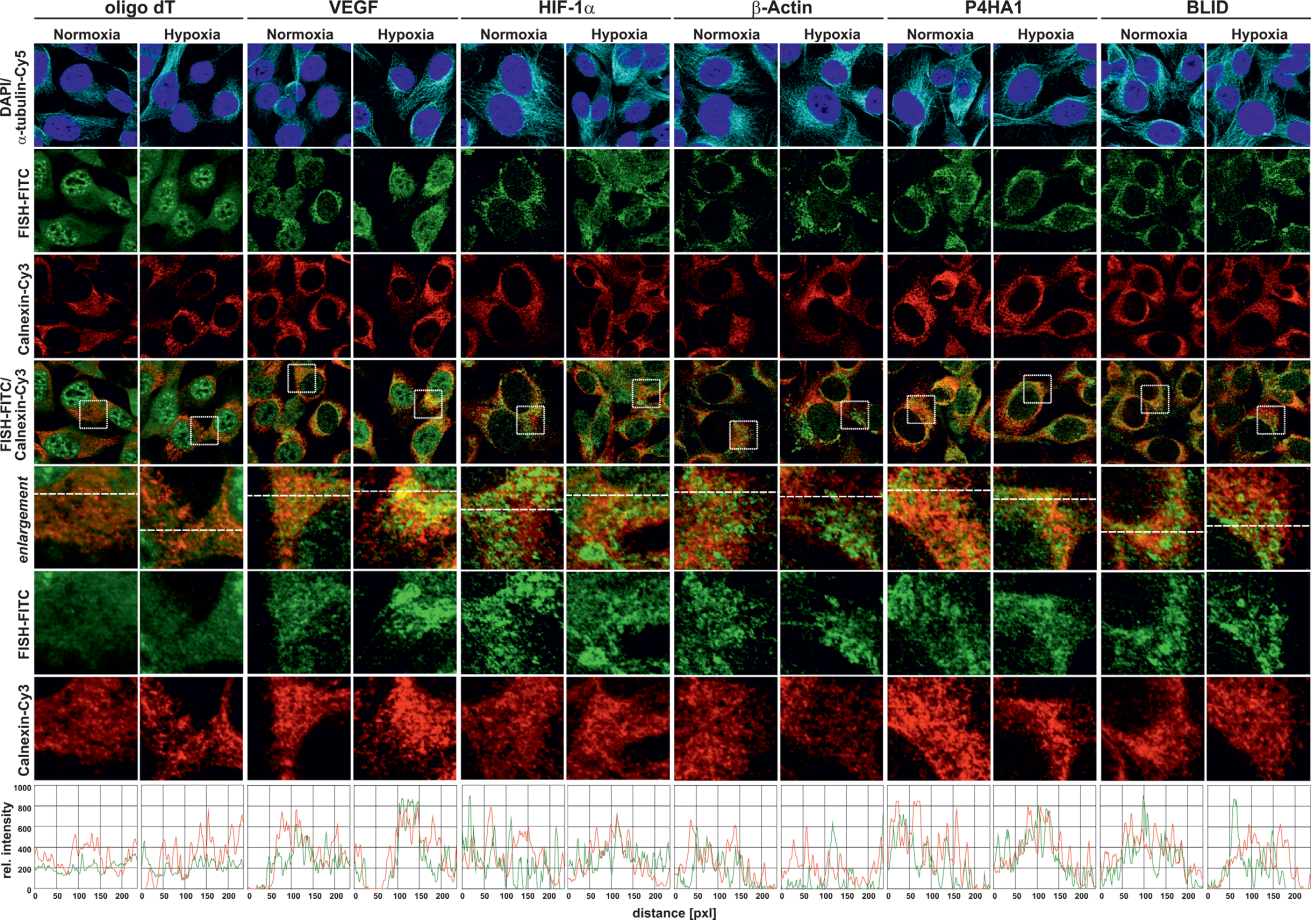
Dual fluorescence detection of transcript co-localization with the ER marker Calnexin by IF-FISH. HT1080 cells were cultured either under normoxic or hypoxic (1% oxygen) conditions for 36 h and hybridized with oligo(dT), VEGF, HIF-1α, β-Actin, P4HA1 and BLID mRNA probes (FITC, green) for *in situ* hybridization. Immunostaining of endogenous α-tubulin (Cy5, cyan) and Calnexin (Cy3, red) was carried out using specific antibodies. The fourth line shows the merged images for co-localization. In the fifth line, dotted squares indicate the magnified area represented in the enlargement. Cy3 and FITC fluorescence monitored along the dashed lines is shown as relative signal intensity in the bottom panel. Co-localization of the transcript with the ER marker Calnexin is indicated by a good match in peaks or troughs of both signals.

To further validate selective transcript localization to the ER, we used immunofluorescence staining and fluorescence *in situ* hybridization (IF-FISH) to visualize cellular transcript localization. Analysis of control cells using a fluorescent oligo(dT) probe revealed typical poly(A) RNA localization as indicated by bright nuclear foci, that excludes the nucleoli, along with a more diffuse staining throughout the cytoplasm (Figure 3). The distribution pattern was largely unchanged in hypoxia, indicating no altered localization for the majority of the transcripts.

We then detected the presence and localization of selected mRNAs by IF-FISH together with co-staining for the ER marker protein calnexin. HIF1A, P4HA1 and the HIF-1 target gene VEGF (vascular endothelial growth factor), all of which are functionally relevant during hypoxia, showed enhanced mRNA co-localization at the ER using Calnexin as a ER-marker (Figure 3), whereas β-Actin and BLID mRNAs were excluded from ER interaction under hypoxic conditions. Of note, to support the view that our selected candidate mRNAs are indeed associated to translation at the ER, we further performed transcript co-staining with the 60S ribosomal subunit protein rpL19 (Supplementary Figure S5). We chose a protein of the 60S ribosomal subunit to avoid detection of non-ribosomal co-localization as the 40S subunit can be part of specific complexes such as stress granules (54,55). Consistantly, we observed that P4HA1, HIF1A and VEGF mRNAs were co-localized with rpL19 during hypoxia, whereas co-localization of oligo(dT), β-Actin mRNA and BLID mRNA was slightly reduced. As a negative control the Golgi marker GM130 was used, which showed no co-localization for any of the RNA probes tested (Supplementary Figure S6). These findings were confirmed in the breast cancer cell line MCF-7 (Supplementary Figures S7–S9) supporting the view that mRNA partitioning during hypoxia is not specific to HT1080 fibrosarcoma cells.

Taken together, our data demonstrate that elevated gene expression of selected candidates in hypoxia is specifically associated to ribosomes at the ER.

### mRNA partitioning is a requisite for hypoxia-induced translation

Next we assessed whether transcript localization at the ER in response to hypoxia was a general mechanism that ensures selective protein synthesis. We hypothesized that transcripts of known functionality in hypoxia, which are not subject to global translation repression, should be elevated at the ER. To test this hypothesis, we performed a microarray-based gene expression analysis comparing RNA from a pure ER fraction as shown in Figure 2, noted here as *ER–RNA*, which accounted for mRNA translation at the ER with *total RNA*, which was used to account for the overall expression level. Candidates whose expression was significantly changed in the microarray were selected by fold-change (FC) and *z*-score (Supplementary Figure S10), as described earlier (42). Verification of microarray data by qPCR of selected candidate mRNAs is shown in Supplementary Figure S11.

**Figure 4.**
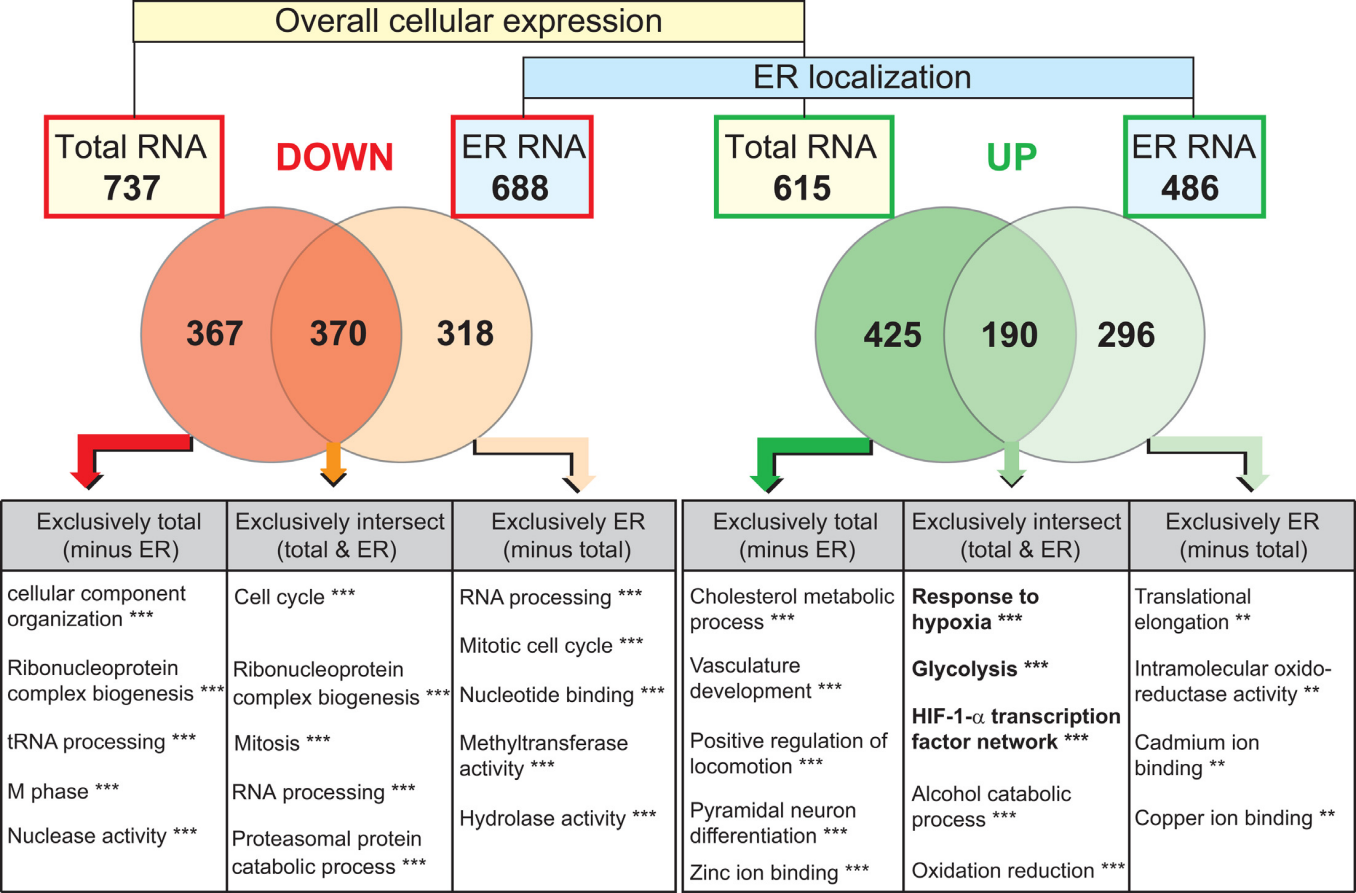
Summary of microarray data analysis. Groups of significantly up- (green) or down- (red) regulated candidates from total RNA (overall cellular expression) or ER-localized RNA (ER localization) were selected. The numbers of candidate genes are indicated in black. Intersections of candidates regulated by both expression level and localization were calculated. Gene ontology analysis was performed using the tools provided by WebGestalt (http://bioinfo.vanderbilt.edu/webgestalt/). For GO analysis only miRNAs and protein coding transcripts were used. **−*P* < 0.01, ***−*P* < 0.001. A detailed description of significantly enriched GOs and statistics is provided in Supplementary Tables S2–S7.

Of the ∼14 800 genes found to be expressed in HT1080 cells, a subset of 1352 genes was either positively (*n* = 615) or negatively (*n* = 737) regulated in hypoxia, while the vast majority of the transcripts (90.8% of expressed genes) remained unchanged (Figure 4). In the ER-RNA group, 1174 genes were either positively (*n* = 486) or negatively (*n* = 688) regulated, indicating that the majority of the transcripts (92.1%) showed no change in ER localization during hypoxia. This finding is in line with the lack of any major change in oligo(dT) distribution that we observed by IF-FISH experiments (Figure 3 and Supplementary Figures S5 to S9 and S15).

Specifically, of the 615 candidates that were upregulated at the total RNA level, only 190 were also increased at the ER. Thus, the majority of 425 genes, despite being upregulated either at the transcriptional level or by increased mRNA stability, showed no increased level in the ER fraction. This suggests that they were not prevented from global suppression of mRNA translation. Strikingly, 296 transcripts showed an increased presence at the ER without being elevated in total. Between transcriptionally-activated genes and transcripts that are regulated by their localization to the ER following hypoxia there was small overlap, which strongly supports a selective subcellular partitioning of the transcriptome between the cytoplasm and ER. Similar observations were made for downregulated transcripts (Figure 4).

To investigate the biological relevance of hypoxia-induced mRNA partitioning, we identified clusters of genes with similar functional properties. Transcripts upregulated at both their expression level and their ER localization were significantly associated with gene GOs such as ‘response to hypoxia’, ‘glycolysis’ and ‘HIF-1α transcription factor network’ (Figure 4). This observation clearly demonstrates that factors crucial for cellular survival are elevated in the ER fraction. These include many HIF target genes, for which numerous studies have shown that they are indeed actively translated during hypoxia. Among the 190 transcripts elevated by total RNA level as well as ER localization, our candidates P4HA1, HIF-1α and VEGF were found. A list of HIF-1 target genes regulated during hypoxia by alteration at the total mRNA level and/ or ER localization in HT1080 fibrosarcoma cells is shown in Supplementary Table S1. Apparently, hypoxia-inducible transcripts, which are not subject to translational repression, are present at the ER where translation persists. Genes that were activated at the transcriptional level, but did not show an increased transcript presence at the ER (i.e. BLID mRNA) were associated with GOs such as ‘vasculature development’, ‘positive regulation of locomotion’ or ‘pyramidal neuron differentiation’. These GOs would be rather expected for the adaptation of endothelial cells, macrophages or neurons to hypoxia, but less for fibroblasts used in our assay. A complete overview of GO analysis including detailed statistics is provided in the Supplementary Tables S2–S7.

Our observations emphasize a specific adaption of gene expression in hypoxia at the level of mRNA translation. Interestingly, functional enrichment analysis of downregulated candidates showed that similar functions are suppressed, independent of whether this occurs at the expression or localization level. This indicates that discrimination of certain mRNAs with respect to ER localization might play a similar role in the regulation of gene expression.

### Transcripts selected for translation at the ER have highly conserved UTRs and varying 5′UTR uAUG contents

Post-transcriptional control of specific transcript subsets by mRNA partitioning between the two compartments of protein synthesis may be mediated by interactions between *cis*-elements and bound trans-factors. Cis-elements are mainly located in the mRNA 5′- and 3′-UTRs (56). Thus, in order to detect possible functional elements we analysed the UTRs of the candidates actively translated at the ER in hypoxia.

**Figure 5.**
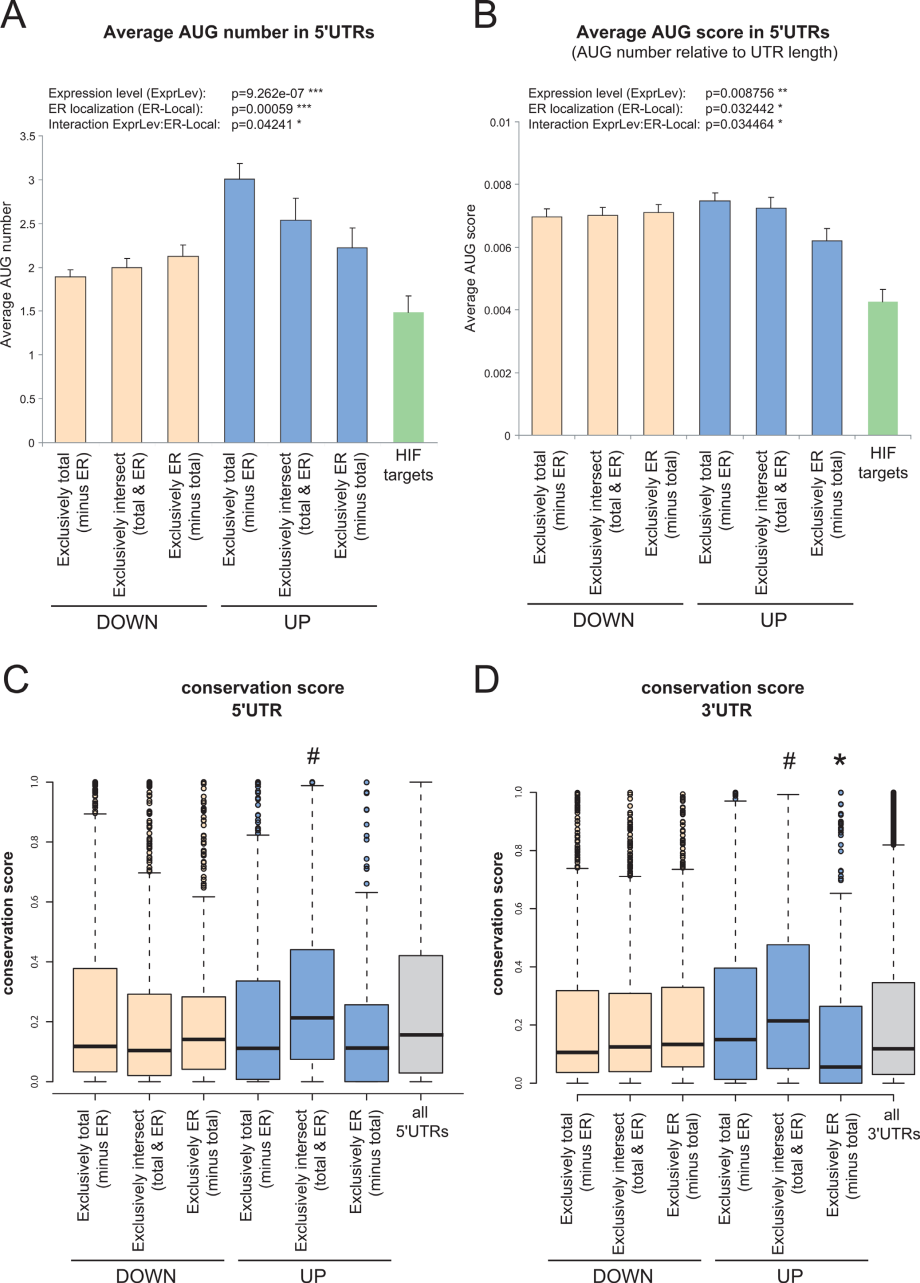
Sub-group specific UTR features. Transcripts of genes that were either up- or downregulated in their expression and/or ER localization as described in Figure 5 were used to test for different UTR features. (**A** and **B**) From all 5′UTR sequences we determined uAUG number (A) and UTR length. The AUG score (B) is the ratio of the number of uAUG and the length of the respective UTR. The gene specific AUG number/score was analysed with a non-parametric two factor ANOVA test with respect to location and expression level. The HIF target gene group consists of 78 verified candidates as described in (1) and is shown for comparison only. (**C** and **D**) Group-specific conservation scores of 5′-UTRs (C) or 3′UTRs (D). A hash sign (#) indicates a significantly higher conservation score compared to all other groups in the statistical post-hoc test. Asterisks (*) indicate a significantly lower conservation score compared to the other groups in the post-hoc test. The grey bars represent either all 5′- or all 3′-UTRs and were not included in the statistical analysis. They are shown for comparison only. A detailed post-hoc statistical analysis is presented in Supplementary Figure S14.

First, we tested whether transcripts of genes regulated during hypoxia show differences in the number of uAUGs in the 5′-UTR as a feature of translational repression during stress (33). Interestingly, transcripts of genes activated during hypoxia assessed by total RNA level alone (exclusively total group) contained more uAUGs than the transcripts of downregulated genes (Figure 5A). This can partially be explained by the significantly longer 5′UTRs in this group (Supplementary Figure S12). Consequently, the uAUG score (mean uAUG number per transcript number relative to UTR length) is also significant, albeit to a lesser extent (Figure 5B). Notably, analysing a set of 78 verified HIF targets as described in (1) indicate that HIF targets in general represent transcripts with less uAUGs (Figure 5A and B). Our observations are further confirmed by a gene-centered analysis of the uAUG score (Supplementary Figure S13) that also indicates a significant interaction with regard to gene expression level and ER localization that depends on uAUGs. These data suggest uAUGs as potential regulatory elements for mRNA translation in hypoxia.

We then focused on the exclusively intersect group (upregulated at expression level and ER localization) representing GOs such as ‘response to hypoxia’ or ‘glycolysis’ and asked first whether the 5′- or the 3′-UTRs display any degree of conservation. Interestingly, both the 5′- and 3′-UTRs in this group showed higher conservation scores when compared to the UTRs in the other groups (Figure 5C, D and Supplementary Figure S14). Moreover, 3′-UTRs of transcripts downregulated in their ER-localization were significantly less conserved and thus might be involved in species-specific regulatory processes (Figure 5D). These data suggest that mRNA partitioning for translation at the ER during hypoxia is an evolutionarily conserved regulatory mechanism, and that mRNA 5′- and 3′-UTRs likely represent common features that mediate escape from the global suppression of translation.

### Enrichment of specific *cis*-elements in mRNA 5′- and 3′-UTRs provide ER localization in hypoxia

To investigate whether hypoxia-inducible transcripts showing ER presence in hypoxia carry specific *cis*-elements in their UTRs, we systematically compared the mRNA 5′- and the 3′-UTRs of the ‘upregulated: exclusively intersect group’ to UTRs with all other transcripts expressed in HT1080 cells. Strikingly, we found significant enrichment of five motifs in the 5′-UTRs and 29 motifs in the 3′-UTRs (Table 1). For functional verification, we selected the top 5′-UTR motif (5′*UTR-cis1*) and two 3′-UTR motifs: (i) the top 3′-UTR motif [3′*UTR-cis1*] and (ii) the motif ranked no. 22 [3′*UTR-cis22*], which showed a high conservation according to the basewise phastCons (44 vertebrate species) conservation score obtained from USCS (http://hgdownload.soe.ucsc.edu/goldenPath/hg18/phastCons44way/). Context analysis by comparing different UTR sequences carrying these motifs indicated similarities in the immediate upstream and downstream regions that were independent of their precise position. Therefore, we cloned ∼40-mer oligonucleotides, bearing each of the selected motifs in the center, into the 5′- or 3′-UTR of the luciferase reporter mRNA as shown in Figure 7A. For the 5′-UTR motif the element 5′-GGGCCACGCTCC***CCGCGC***CTCGGCTTCGCG-3′ was derived from the EGLN3 mRNA 5′-UTR. The 3′-UTR elements were obtained from the PLOD2 mRNA (3′*UTR-cis1*: 5′-GCATTTAATTATTT***TTTAAAAA***ACTTTTTAAGTACTTGAA-3′) and CA9 mRNA (3′*UTR-cis22*: 5′-TCCTGTCCTGCTCATT***ATGCCA***CTTCCTTTTAACTGCCAA-3′), respectively. For each motif, its corresponding mutant was cloned accordingly. Cells transiently transfected with the various wild-type and mutant constructs were analysed for both (i) transcript localization and (ii) expression levels under control and hypoxic conditions.

**Figure 6.**
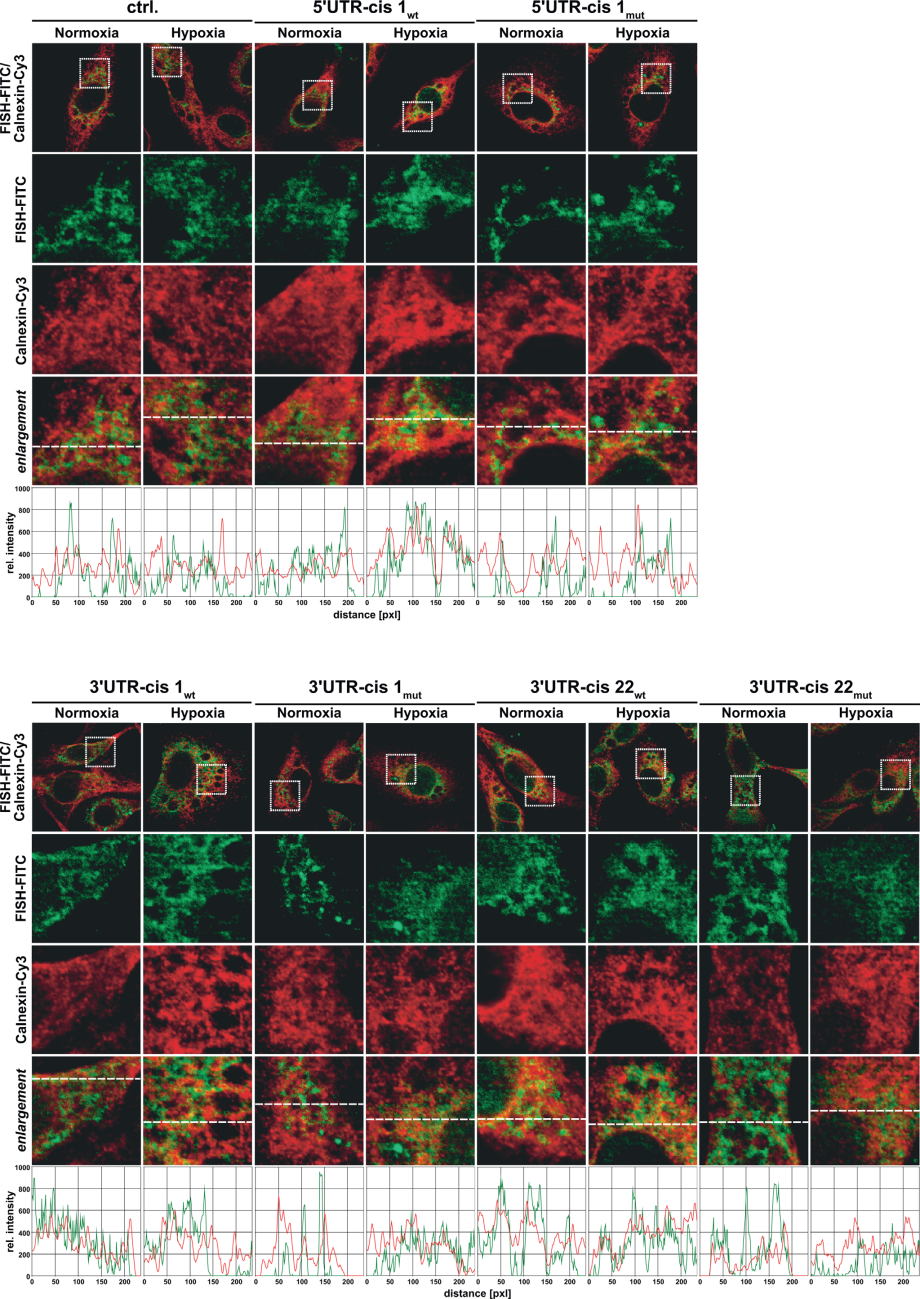
IF-FISH analysis of HT1080 cells transfected with Firefly-Luciferase (Luc) reporter plasmids by electroporation as indicated and cultured under normoxic or hypoxic conditions. Cells were hybridized with Luc mRNA probe (FITC, green). The native Luciferase mRNA is shown as control (ctrl.). Modified Luciferase transcripts contained insertions of 5′- or 3′-UTR *cis*-elements as shown in Table 1, with either the original sequence context (wt) or a mutated *cis*-Element (mut) as shown in Figure 7A. Nuclei were stained with DAPI (blue). Specific antibodies were used to co-stain endogenous a-tubulin (Cy5, cyan) and Calnexin (Cy3, red). Dotted squares indicate the magnified area represented in the enlargement. Cy3 and FITC fluorescence monitored along the dashed lines is shown as relative signal intensity in the bottom panel.

**Figure 7.**
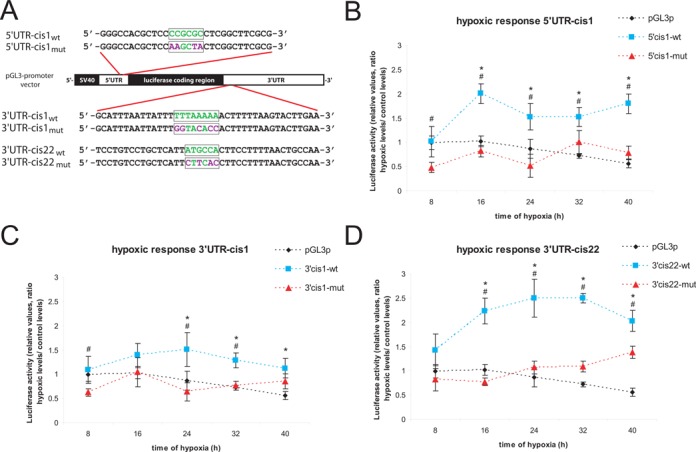
Verification of motif functionality by reporter gene assays. The pGL3-promoter vector (SV40 promoter) was used to insert 30–40-mers carrying selected motifs (boxes) in its center into the 5′- or 3′-UTR of luciferase mRNA. (**A**) Schematic illustration of cloning strategy and inserted sequences. Native motifs (wt) and mutated variants (mut) were tested. (**B**–**D**) HT1080 cells were transfected and incubated for up to 40 h under control or hypoxic conditions. Relative fold changes as compared to control conditions are shown for the original pGL3-promoter construct as well as constructs carrying native or mutated *cis*-elements in the luciferase 5′UTR (5′UTR-cis1) or 3′UTR (3′UTR-cis1 and 3′UTR-cis22). *n* = 8. * indicates significantly changed luciferase activity of the wt-construct compared to the unmodified luciferase mRNA (pGL3p) and # indicates significance compared to the mutated variant (*P* < 0.05).

**Table 1.** Motif enrichment in 5′- and 3′-UTRs of candidates that are increased in total mRNA level and being increasingly present at the ER during hypoxia in HT1080 cells

Type	Motif	*P*-value	*E*-value	Examples carrying the motif in its UTR
5′UTR				
1	C[C,G,A]GCGC	1.70E-13	5.30E-09	EGLN3, HK2, GPI, ENO2, PFKFB4, PDK1
2	CCGCCC[C,G,T]C	5.60E-13	1.60E-08	HIF1A, EGLN1, HK2, CITED2, NDRG1, P4HA1
3	ACG[A,C,T]GC	2.50E-11	7.30E-07	ITPR1, ENO2, HK2, EGLN1, PLOD2, AOX1, GPI
4	C[C,G]AC[C,T]C	5.10E-11	1.40E-06	CA9, PDK1, ANG, SMAD9, VLDLR, LOX, PGM1
5	GCGGG[C,A]	1.10E-09	3.10E-05	ARG2, ANG, VLDLR, STC2, EGLN1, PDK1
3′UTR				
1	TTTA[C,G,A]AAA	2.40E-16	1.10E-11	HIF1A, PFKFB4, PGK1, HK2, P4HA1, PLOD2
2	[C,A]TG[A,T]AAA	4.70E-16	2.10E-11	MMP-9, VEGFA, PFKFB4, PFKM, PGK1, HK2
3	GG[A,G,T]TAA	6.80E-14	3.00E-09	PGM1, ARG2, PLOD1/2, ITPR1, PAM, SMAD9
4	A[C,A]TAGA	8.10E-14	3.50E-09	PFKFB4, PRKAA2, GPI, ENO2, PGK1, HK2
5	A[A,G,T]AAATC	3.30E-13	1.40E-08	GPI, HK2, PGK1, KLF5, CITED2, LOX, PRKAA2
6	AAAA[C,T]A[C,T]	4.00E-13	1.70E-08	ALDOC, SMAD9, PLOD2, ITPR1, P4HA1, STC2
7	TAGC[A,G,T]A	8.60E-14	3.70E-09	HK2, PGK1, SMAD9; ALDOC, PLOD2, ITPR1
8	A[C,A]TTGTG	6.70E-13	2.90E-08	PRKAA2, ENO2, PGK1, HK2, BNIP3, EGLN1
9	TTTAAG[A,T]	9.60E-13	4.20E-08	ARG2, P4HA1, PLOD2, ITPR1, SMAD9, STC2
10	A[A,G]AGAAT	7.80E-13	3.30E-08	ARG2, BNIP3, SMAD9, EGLN1, GPI, NOX4
11	AATGT[C,A]	1.30E-12	5.60E-08	PFKFB4, PRKAA2, GPI, PFKM, PGK1, HK2
12	[C,G,T]T[A,G]TTTG	3.50E-12	1.50E-07	ENO2, PGK1, HK2, PLOD2, EGLN1, PFKFB4
13	AAAAAAA[A,G]	5.60E-12	2.40E-07	CITED2, PLOD2, SMAD9, ITPR1, GPI, ERO1L
14	TAA[T,G]C[A,T]	3.00E-13	1.30E-08	ENO2, HK2, PGK1, PFKM, BNIP3, PAM, PLOD2
15	AAAT[C,G]T	3.30E-12	1.40E-07	P4HA1, EGLN1, ARG2, ANG, NDRG1, PAM
16	TA[T,G]GAA	2.30E-12	9.50E-08	PLOD2, ITPR1, P4HA1, EGLN1, PAM, CITED2
17	[C,G,T]TTTA[A,T]A	4.30E-12	1.80E-07	CA9, CITED2, BNIP3, PGK1, HK2, GPI, PRKAA2
18	ACT[A,C,T]AC	1.20E-12	4.90E-08	PRKAA2, ENO2, PGM1, HK2, GLRX, ITPR1
19	TGCAC[A,T]	7.10E-12	2.90E-07	PFKFB4, PLOD1, PGK1, PFKM, NDRG1
20	ATA[C,T]A[C,G]	6.60E-12	2.70E-07	ALDOC, PRKAA2, HK2, PGK1, BNIP3, ARG2
21	A[A,T]ACTG	1.00E-11	4.00E-07	P4HA1, EGLN1, ARG2, PAM, NDRG1, GPI, HK2
22	AT[C,A]CAA	1.20E-11	5.00E-07	HIF1A, ENO2, EGLN3, CA9, PRKAA2, GPI
23	A[A,T]GAGA	2.30E-11	9.10E-07	ARG2, PLOD1/2, STC2, PAM, PFKFB4, HK2
24	AACAC[T,G]	3.00E-11	1.20E-06	P4HA1, GPI, HK2, PLOD2, GLRX, MT1E, MT1F
25	AACTAT	4.90E-08	1.90E-03	GLRX, ERO1L, LOX, SMAD9, CITED2, STC2
26	AACCAA	5.90E-08	2.30E-03	PLOD1, SMAD9, ARG2, P4HA2, GPI, PGK1
27	AATAAAT	2.30E-08	9.10E-04	ALDOC, CA9, EGLN1, BNIP3, GPI, PRKAA2
28	AAAGGCA	2.50E-07	9.60E-03	SMAD9, STC2, ERO1L, BNIP3L, AOX1, GPI
29	GTTTACA	4.30E-09	1.70E-04	PFKFB4, PRKAA2, P4HA1, LOX, PGM1, PGK1

Alternative nucleotides are shown in squared brackets. Gene symbols of genes that bear the motifs in its mRNA UTRs are listed exemplarily.

IF-FISH analysis indicated that two out of the three motifs tested (motifs: 5′*UTR-cis1* and 3′*UTR-cis22*) did indeed mediate increased luciferase transcript localization at the ER as a result of hypoxia (Figure 6), while the mutated elements failed to show this translocation. The top 3′-UTR element (3′*UTR-cis1*) showed only weak increased ER co-localization upon hypoxia, however, compared to the native luciferase transcript we observed increased ER localization under control conditions. Thus, we suggest that this motif mediates ER localization *per se*. IF-FISH data for oligo(dT) and mock transfection are provided in Supplementary Figure S15.

Functional verification by reporter gene assays confirmed that the *cis*-elements that mediated ER localization in turn resulted in elevated gene expression during hypoxia (Figure 7). During a time course of up to 40 h, we found that all of the selected motifs enhanced expression in hypoxia relative to their corresponding mutants (Figure 7B-D). Of interest, the motif 3′*UTR-cis1*, which confers increased ER localization *per se*, showed only a weak activation in hypoxia. However, compared to the native luciferase transcript, insertion of the ER-localization element led to an elevated and stable expression rate from short to long term hypoxia, supporting the view that transcripts localized at the ER are less affected by global suppression of protein synthesis in hypoxia.

In line with 5′- and 3′-UTR conservation of transcripts that are increased in hypoxia at the mRNA level and ER localization (upregulated: exclusively intersect group [total and ER]), these mRNAs show clustering of specific motifs in both UTRs. The *cis*-elements tested here either provide ER transcript localization *per se* or under hypoxic conditions. In both cases these *cis*-elements mediate sustained or elevated protein synthesis under conditions of global suppression of mRNA translation.

## DISCUSSION

Oxygen depletion alters gene expression to facilitate cell survival until oxygen supply is restored. The cellular adaptation to the changed environment requires the repression of energy-consuming processes, especially protein synthesis, while activating the expression of survival factors.

Many efforts have been undertaken to explain the escape of specific transcripts from global suppression of 5′-cap-dependent initiation of mRNA translation during hypoxia. These studies focused on the cytoplasmic compartment for protein synthesis. In this study we correlated gene expression rates for selected candidates, as assessed by total RNA and protein levels, with mRNA localization in the subcellular compartments relevant for mRNA translation, namely (i) translationally-inactive RNP complexes, (ii) transcripts associated with cytoplasmic ribosomes and (iii) transcripts translated at the ER. Based on findings employing P4HB, P4HA1, HIF-1α, BLID and β-Actin mRNAs, which represent both hypoxia-inducible and non-inducible candidates, we hypothesized that mRNA translation is regulated by different mechanisms that act either in the cytoplasm or at the ER. Subsequently, we showed that the increased gene expression for specific hypoxia-inducible candidates is associated with increased transcript localization to the ER. In line with the observation that the spatial organization of mRNA translation between the cytoplasm and the ER represents a regulatory mechanism during stress (34), our findings highlight the ER as a crucial compartment ensuring active mRNA translation during hypoxia.

Specifically, using the unfolded protein response (UPR) as a means to physiologically modulate mRNA translation, it has been reported by Stephens *et al*. and Reid *et al*. that the ER serves as the preferred site for the synthesis of both cytosolic and signal sequence-bearing proteins (57,58). They showed that mRNAs that underwent translation on both cytosolic and ER-bound ribosomes displayed ER-restricted loading into polyribosomes in response to UPR induction. They concluded that the capacity to regulate protein synthesis differentially in the cytosol and ER compartments allows the ER to serve as a privileged site of protein synthesis (57). Consistently, infection with Coxsackie virus B3 to mediate suppression of cellular protein synthesis also showed retained translational activity in ER-bound ribosomes (34,57). Thus, our findings highlight those previous observations while complementing them, using different techniques and strategies to study the influence of hypoxia as a physiological stimulus to inhibit global protein synthesis. Interestingly, both the conditions of UPR modulation and infection by Coxsackie virus B3 resemble hypoxia as they also result from an inhibition of 5′-cap-dependent initiation of mRNA translation. Altogether, these findings indicate that the selection of transcripts for translation at the ER, under conditions of global suppression of protein synthesis, is a fundamental and conserved process to ensure adaptation of gene expression to stress situations such as hypoxia.

Our data indicate that mRNA partitioning between the cytoplasm and the ER during hypoxia is highly selective. In a global setting we observed that only a few of the candidates that were up- or downregulated at the expression level were also increasingly or decreasingly present at the ER. For instance, in our setting 615 genes were upregulated in response to hypoxia. Only 190 (31%) thereof were also elevated in the ER fraction. Factors upregulated by expression and ER localization belong to the hypoxia-relevant GOs ‘hypoxia response’, ‘HIF-1α transcription factor network’ and ‘glycolysis’. As factors belonging to these categories are well known to be translated under hypoxic conditions, this observation strongly supports the view of hypoxia-dependent selective subcellular partitioning of the transcriptome for active mRNA translation at the ER.

Our results further indicate that mRNA partitioning might be a filter for cell type-specific adaptation to hypoxia. Not all genes that are activated at the transcription level are necessarily translated in every cell type. Only those that are essential to providing a cell-type appropriate response to hypoxia are likely to be translated. For example, transcripts that belong to the GO ‘pyramidal neuron differentiation’ are not elevated at the ER in the fibroblasts used in this study, suggesting that they are subject to global translational repression. Future studies are required to show whether the transcript subpopulations that are increased at the ER in hypoxia differ between cell types. Nevertheless, factors that maintain energy metabolism and thus are likely to be regulated by similar mechanisms in different cell types during hypoxia, are translated favourably at ER associated ribosomes as shown by transcript co-localization with the ER marker calnexin and the ribosomal protein rpL19 in this study.

At present, our understanding of mRNA tethering to the ER independent of the signal-recognition particle is quite limited, and the identification and functional characterization of mRNA receptors as well as their dynamics require further investigation (58). Highly conserved regions within mRNA UTRs, which function as stress sensors, indicate a crucial function of mRNA UTRs in the regulation of gene expression (59,60). Consistently, we found that mRNA 5′- and 3′-UTRs of candidates that are induced at the mRNA level and favourably associate with the ER in hypoxia showed a higher conservation score, indicating that transcript selection for sustained translation in hypoxia is an evolutionarily conserved process. Indeed, previous experiments suggested the existence of ER-localization signals to be present within the mRNA molecules themselves (61). Moreover, it has been hypothesized that functionally related genes are regulated as groups by specific mRNA-binding proteins to form post-transcriptional operons (62). This might involve sequence motif recognition-dependent paths that utilize *cis*-encoded localization information (zip codes), and both known and unknown RBPs as ER-targeting factors (63). It seems likely that ER-associated mRNAs do not passively diffuse to ER membranes, but might instead arrive via active transport (64). Moreover, analysis of ER membrane proteins revealed that they possess stretches of basic residues in cytosol-facing loops that could have selective RNA binding activity (65).

We found that upstream translation initiation codons are significantly enriched in 5′-UTRs of genes that are upregulated in hypoxia. It is interesting that hypoxia inducible transcripts that are also increasingly present at the ER in hypoxia contain less uAUGs and, thus, do not follow this trend. Moreover, HIF targets in general contain significantly lower numbers of uAUGs, in line with effective translation during hypoxia, as uAUGs have been associated with suppressed translation rates (33). Paradoxically, it has been shown that some transcripts with uORFs are upregulated in response to eIF2α phosphorylation (66). For instance, CITED2 (67) and ATF4 (68) exemplify two uORF-containing transcripts for which the expression is induced by enhanced mRNA translation following stress (66). However, these examples and our findings support the view that uAUGs are regulatory elements that are involved in the control of mRNA translation in the cytoplasm rather than at the ER.

Strikingly, we found enrichment of specific motifs in the 5′- and 3′-UTRs of transcripts, for which elevated expression and increased localization at the ER was detected under prolonged hypoxia. We identified 34 motifs (5 in the 5′-UTRs and 29 in the 3′-UTRs). The functionality of these motifs was exemplarily confirmed by insertion into the luciferase mRNA 5′- or 3′-UTR followed by verification of *cis*-element-mediated ER localization and subsequent expression level. The identified mRNA 5′-UTR motifs are CG-rich and the 3′-UTR motifs are mainly AU-rich. Of note, the HIF-2α–RBM4–eIF4E2 complex binds in close proximity to CGG tri-nucleotides (28). Also, the protein DRBP76 binds to an AU-rich stem loop to enhance the HILDA complex formation that in turn directs the RNA switch and promotes mRNA translation (30). However, 5′-UTRs in general have a tendency to be CG-rich, 3′-UTRs to be AU-rich. We speculate that the motifs identified in our study are core binding sites for *trans*-acting factors and that elements up- and downstream of the respective motif may impair their functionality. Moreover, the number and presence of multiple copies of these elements may have an additional effect on gene expression. Out of the motifs presented in this study, the number of these *cis*-elements per mRNA is high in hypoxia-inducible candidates [HIF1A: *n* = 31, P4HA1: *n* = 32, HK2: *n* = 54 VEGFA: *n* = 32]. In contrast, ACTB mRNA bears *n* = 13 and BLID mRNA only *n* = 7 of these *cis*-elements. Since just one of these motifs is sufficient to enhance ER localization and gene expression in an experimental setting, it is likely that a combination of these motifs as well as effects of other (maybe inhibitory) elements in the native mRNA will have a cumulative effect, which then determines the net functional impact on ER localization and, thus, gene expression. This perspective would further explain differences in the efficacy of specific mRNA-ER localization in hypoxia. Therefore, we hypothesize that clustering of mRNA 5′- as well as 3′-UTR *cis*-elements is pivotal for proper mRNA partitioning to the ER, which represents a mechanism for adaptation of gene expression during hypoxia, especially for HIF-target genes.

Nevertheless, it has been demonstrated that specific mRNAs may be continuously associated with cytoplasmic polysomes in hypoxia (53). Therefore, analysing specific control of mRNA translation in hypoxia means taking into account all subcellular compartments such as the cytoplasm and the ER as well as mRNP complexes. Obviously, the site of translation initiation seems to be a crucial determinant (69) and might hint at the underlying mechanism. It is, for instance, likely that 5′-cap-dependent inhibition affects cytoplasmic translation initiation, thus, favouring IRES-dependent translation, while at the ER 5′-cap-dependent initiation is still active. However, in a hypothetical scenario, IRES trans-acting-factors (ITAFs) may also act as mediators for ER localization. At present, we cannot rule out whether different mechanisms such as HILDA induced RNA-switches, eIF4E1-dependent mechanisms, HIF-2α–RBM4–eIF4E2 complex binding or IRES/ITAF-mediated mechanisms also participate in mRNA partitioning to the ER or whether these pathways represent independent options to prevent global suppression of mRNA translation in hypoxia. We assume that different mechanisms exist and function cooperatively and that candidate selection for ER translation during hypoxia does not act as an on and off switch. Rather, translation at the ER is favoured under specific conditions. Future studies need to address the localization and functionality of *trans*-factors such as RBPs as well as mRNA recognition by ribosomes at the ER. We propose that the clustering of mRNA *cis*-elements is a crucial determinant of mRNA localization in hypoxia.

Our data show that mRNA translation at the ER provides an option to select transcripts for effective protein synthesis under hypoxic conditions. However, the underlying reason for protein synthesis at ER-associated ribosomes during hypometabolism is still unknown. When energy production is diminished, energy consuming transport processes along the cytoskeleton are probably reduced, causing suppression of protein synthesis in the cytoplasm. Interestingly, perinuclear clustering of mitochondria following hypoxia has been described (70) and ER-mitochondria-junction has been implicated in ROS and calcium signalling (71,72). However, the low *K*_m_ of the mitochondrial cytochrome oxidase dictates that virtually all oxygen is consumed by the mitochondria under hypoxic conditions (73). Therefore, gathering mitochondria around the nucleus, and thus the ER, may reflect the recruitment of adenosine triphosphate producers to the vicinity of protein synthesis. Forced by limited cellular energy supply, cells may minimize the active cellular compartment by restricting metabolic processes to the perinuclear zone.

During hypoxia, spatial re-organization of metabolic processes, including mRNA translation, requires tight regulatory control. mRNA partitioning can represent a crucial check-point to divide protein synthesis between the two subcellular compartments, the cytoplasm and the ER. Our data support the view that both compartments are under distinct regulatory control and that translation at the ER is favoured for HIF-target genes and thus contributes to maintain energy metabolism in hypoxia. The selection of transcripts for translation at ER-associated ribosomes seems to be mediated by *cis*-elements located in both the mRNA 5′- and 3′-UTRs. Analysing the alteration of gene expression during oxygen deprivation thus needs to include investigations on mRNA partitioning to select transcripts for protein synthesis at the ER. Future studies will need to address the mechanisms of mRNA partitioning by *cis*-element/ *trans*-factor interaction during hypoxia.

## SUPPLEMENTARY DATA

Supplementary Data are available at NAR Online.

SUPPLEMENTARY DATA
