# Supplementary material for: Hypoxia-induced gene expression results from selective mRNA partitioning to the endoplasmic reticulum
Source: Nucleic Acids Res. 2015 Mar 8;43(6):3219–36. doi: 10.1093/nar/gkv167 (PMC4381074; doi:10.1093/nar/gkv167)
Supplement: SUPPLEMENTARY DATA [file supp_43_6_3219__index.html]

Hypoxia-induced gene expression results from selective mRNA partitioning to the endoplasmic reticulum — Hypoxia-induced gene expression results from selective mRNA partitioning to the endoplasmic reticulum — SUPPLEMENTARY DATA 

# Hypoxia-induced gene expression results from selective mRNA partitioning to the endoplasmic reticulum

## SUPPLEMENTARY DATA

**Files in this Data Supplement:**

- SUPPLEMENTARY DATA
