# Supplementary material for: Hypoxia-induced gene expression results from selective mRNA partitioning to the endoplasmic reticulum
Source: Nucleic Acids Res. 2015 Mar 8;43(6):3219–36. doi: 10.1093/nar/gkv167 (PMC4381074; doi:10.1093/nar/gkv167)
Supplement: SUPPLEMENTARY DATA [file supp_gkv167_nar-00250-v-2015-File009.pdf]

SUPPLEMENT

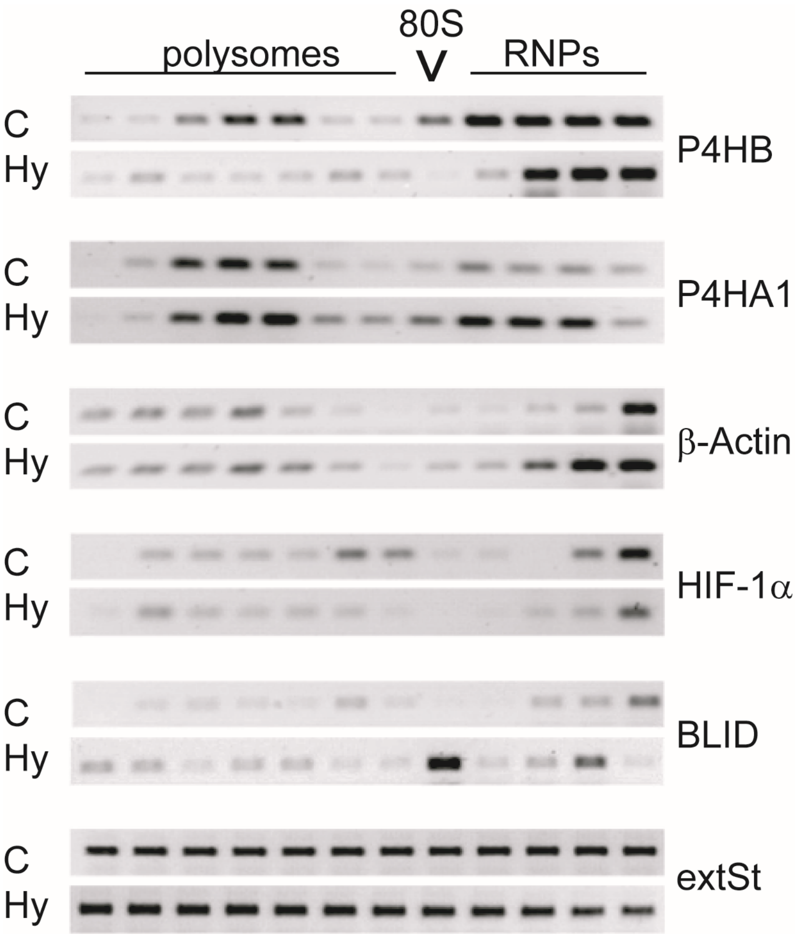

Figure S1: Polysomal gradient analysis. HT1080 cells were incubated under control (21% oxygen) or hypoxic (1% oxygen) conditions for up to 36 h as described in Figure 1. Shown are representative original RT-PCR data (30 cycles for β-Actin and the external standard [extSt]; 35 cycles for the other genes) from pooled samples to visualize mRNA distribution following fractionation of sucrose gradients at control conditions (C) and 36 h of hypoxia (Hy). The external standard (a synthetic *in vitro* transcript) was diluted to appropriate concentration for qPCR and added directly after gradient fractionation and prior to RNA isolation as a technical control. The external standard served for fraction dependent normalization.

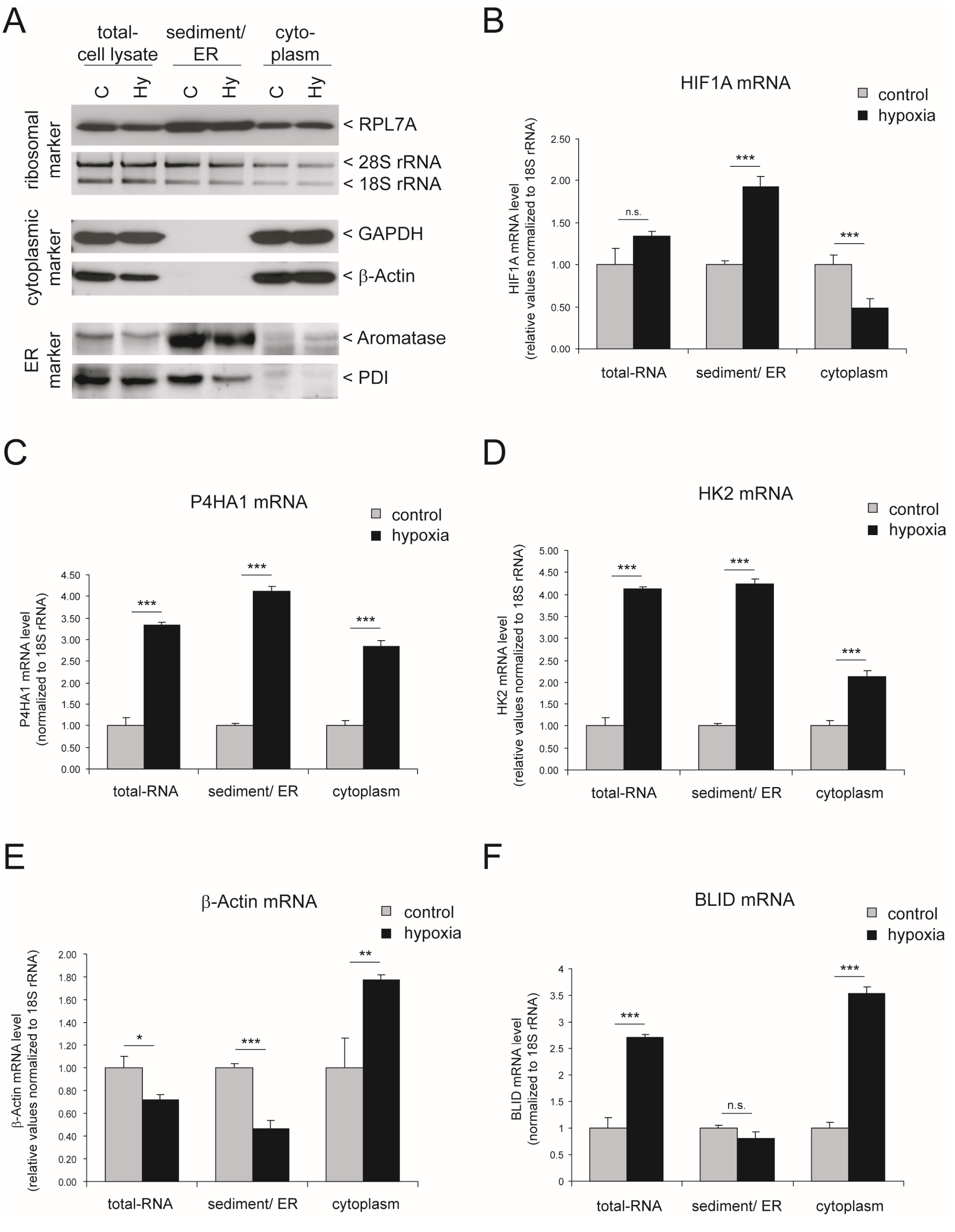

Figure S2: Expression of mRNA candidates in ribosomes-containing sub-compartments. A: Representative Western blot analyses of markers in the sub-compartments. S10 supernatants as used in Figure 1 for polysomal gradient analysis represent a cytoplasmic fraction as demonstrated by cytosolic markers (GAPDH, β-Actin). Both, cytoplasmic and sediment fractions contain polysomes as shown by ribosomal markers (28S and 18S rRNA, ribosomal protein RPL7A). Sediments, however, contain ER as indicated by the presence of the ER marker proteins (PDI and aromatase). **B-F**: Histogram plotting qPCR analyses of mRNA candidates in the various cellular compartments. Expression of B: hexokinase-2 (HK2), C: HIF-1α (HIF1A), D: β-Actin, E: collagen prolyl-4 hydroxylase α(I) (P4HA1) and F: BLID mRNAs is shown. HK2, HIF-1α and P4HA1 were chosen as markers of active translation in hypoxia and, thus, were expected to be present in actively translating ribosomes. Cells were cultured under control (grey) and hypoxic conditions (solid black). N=6. n.s. – not significant. *-p<0.05, **-p<0.01, ***-p<0.001.

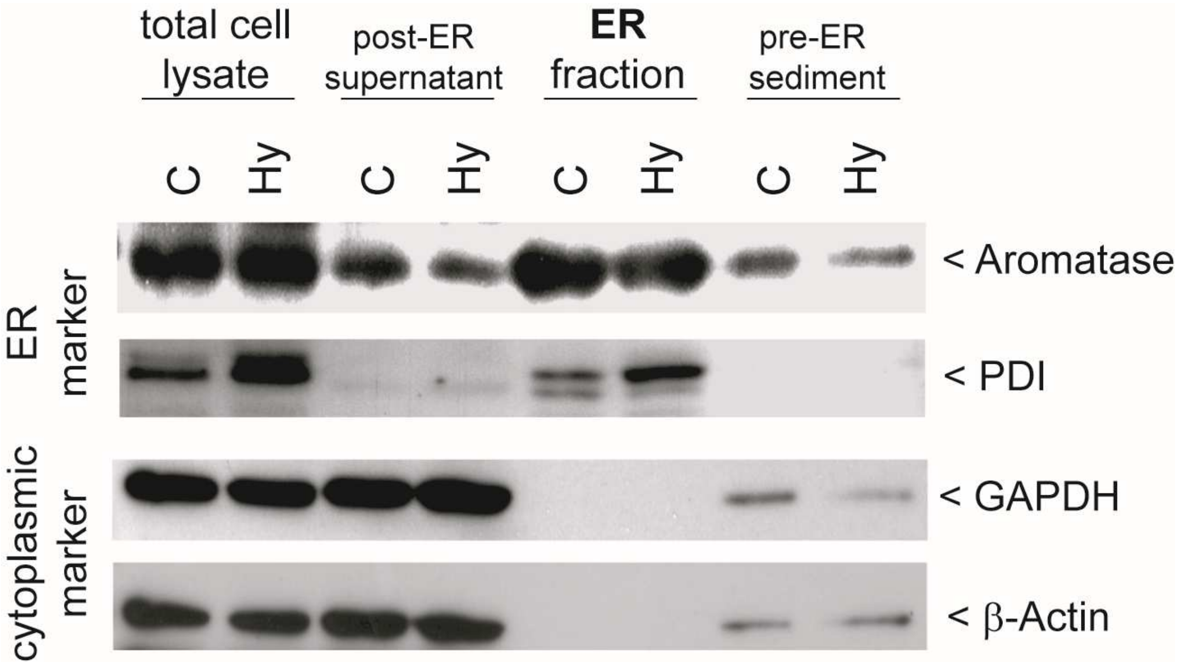

Figure S3: Estimation of marker protein levels in the ER fraction obtained by the SIGMA ER-isolation kit. HT1080 cells were incubated under (C) control (21% oxygen) or (Hy) hypoxic condition for 36 h. A pure ER fraction as shown in Figure 2 and as used for micro-array analysis (Figure 4) was isolated by using the SIGMA ER-isolation kit according to the manufacturers recommendations. Detection of aromatase and protein disulfide isomerase (PDI) served as ER marker proteins. As cytosolic markers GAPDH and β-Actin were used.

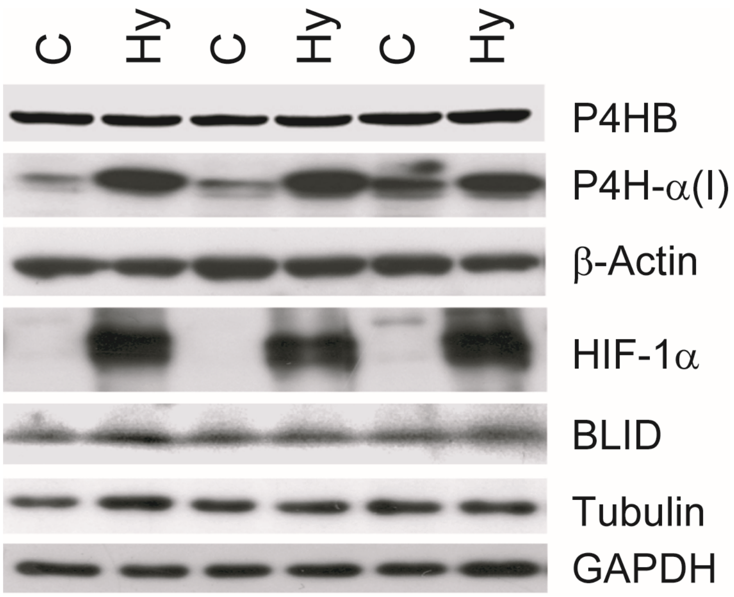

Figure S4: Detection of protein levels for selected candidates. HT1080 cells were incubated under (C) control (21% oxygen) or (Hy) hypoxic (1% oxygen) conditions for 36 h. Representative original Western Blot results are shown to indicate protein levels for candidates used in this study. Detection of relative tubulin levels served as loading control.

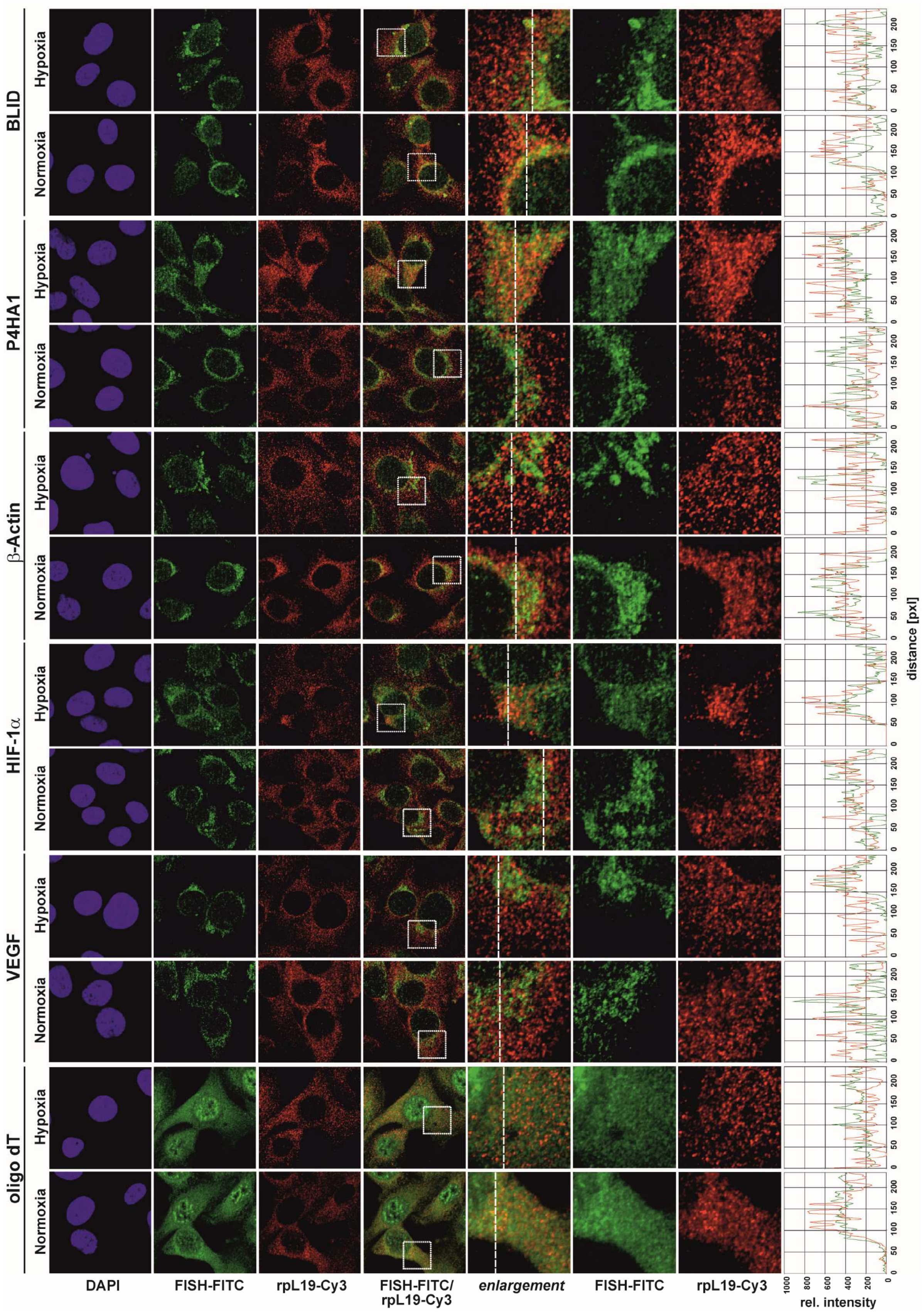

Figure S5: IF-FISH analysis: Co-staining of specific transcripts with the ribosomal marker rpL19. HT1080 cells were treated and analyzed as described in Figure 3. Co-localization of the transcript with the ribosomal marker rpL19 is indicated by a good match in peaks (either up or down) of both signals (bottom line). rpL19 was chosen as it is part of the large ribosomal subunit and, thus, is not associated with stress granules.

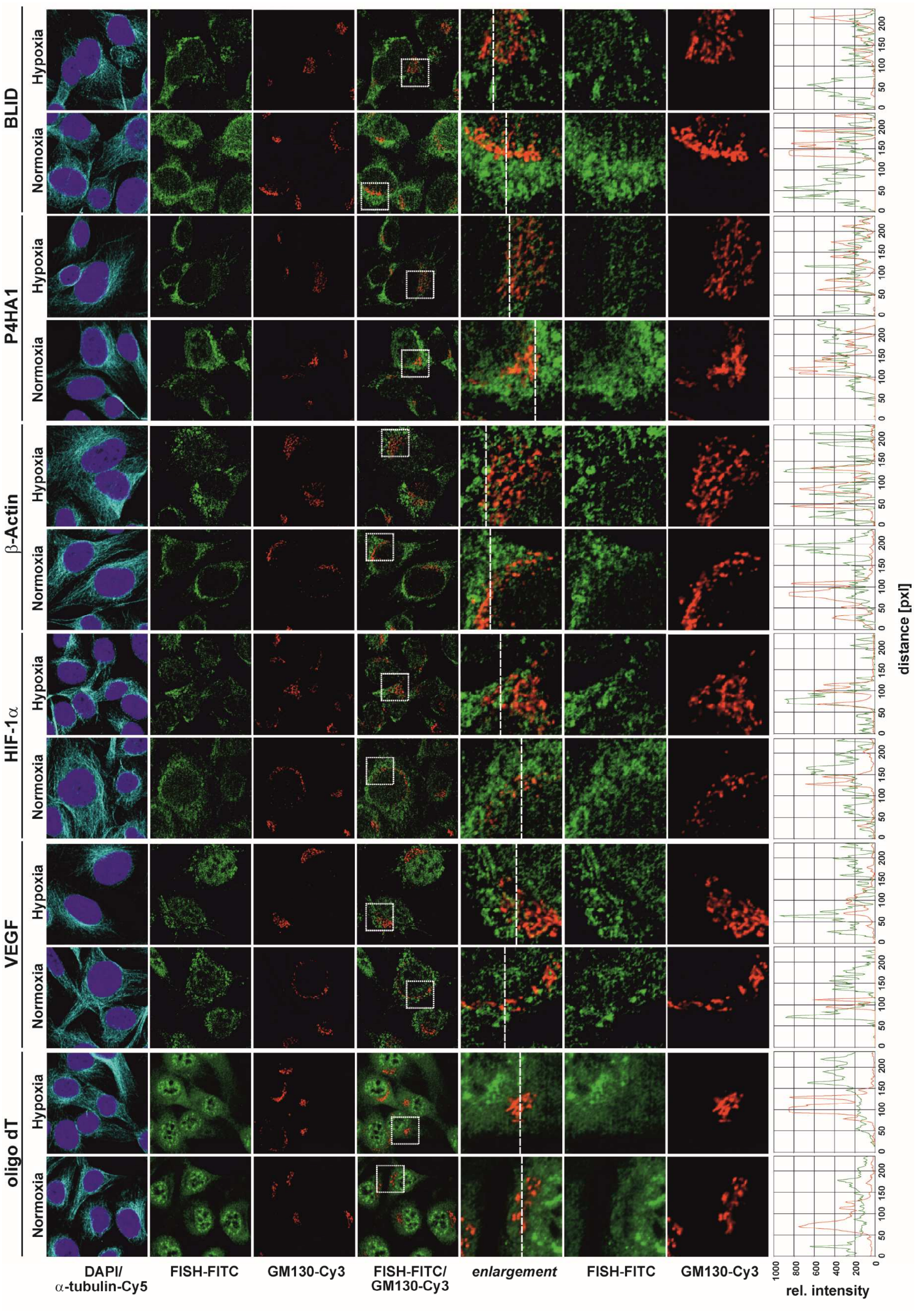

Figure S6: IF-FISH analysis: Co-staining of specific transcripts with the golgi marker GM130. HT1080 cells were treated and analyzed as described in Figure 3. Co-localization of the transcripts with the golgi marker GM130 would be indicated by a good match in peaks (either up or down) of both signals. However, no signal overlap is observed.

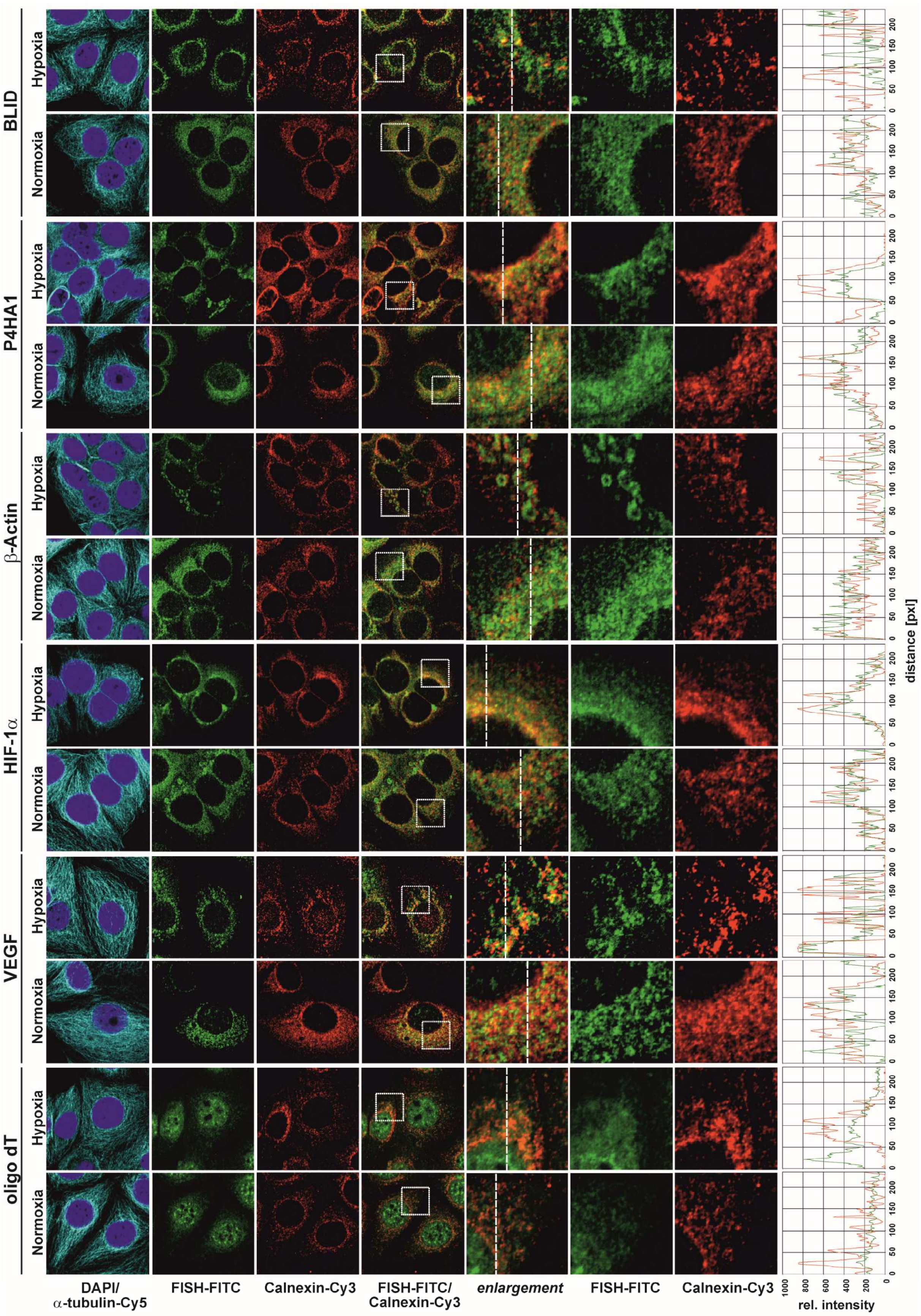

Figure S7: Dual fluorescence detection of transcript co-localization with the ER marker Calnexin by IF-FISH. MCF-7 cells were treated and analyzed as described in Figure 3.

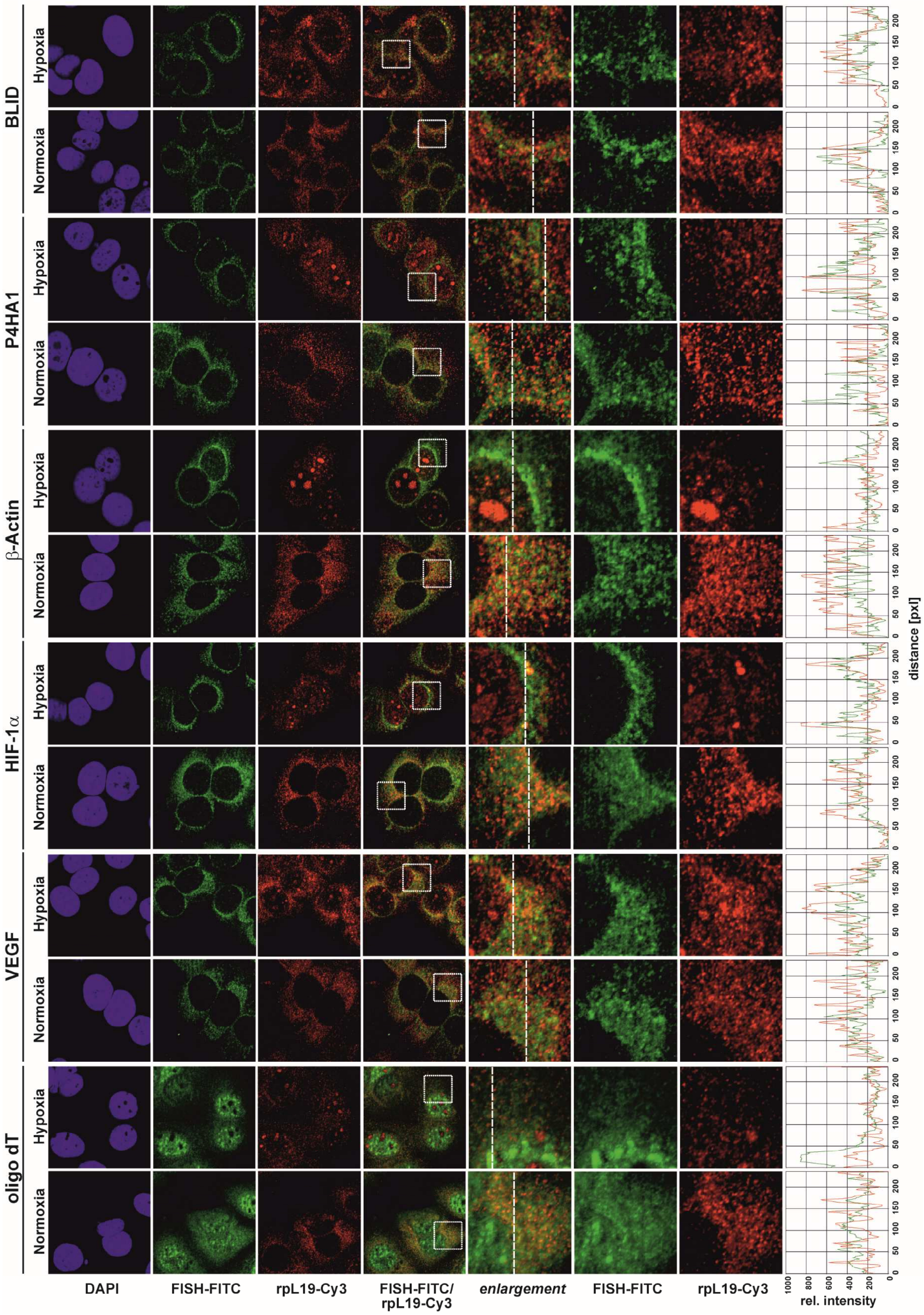

Figure S8: IF-FISH analysis: Co-staining of specific transcripts with the ribosomal marker rpL19. MCF-7 cells were treated and analyzed as described in Figure 3.

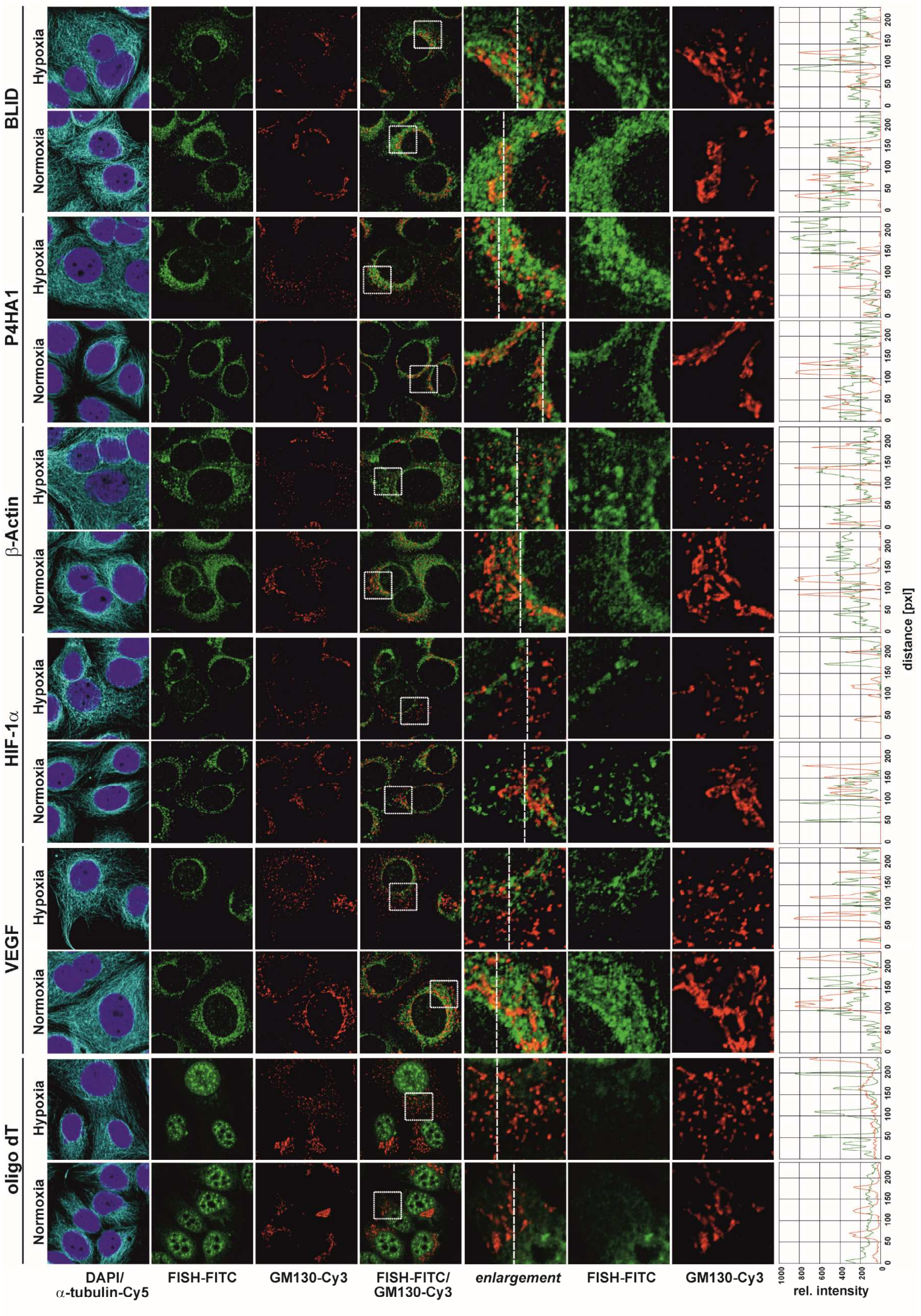

Figure S9: IF-FISH analysis: Co-staining of specific transcripts with the golgi marker GM130. MCF-7 cells were treated and analyzed as described in Figure 3.

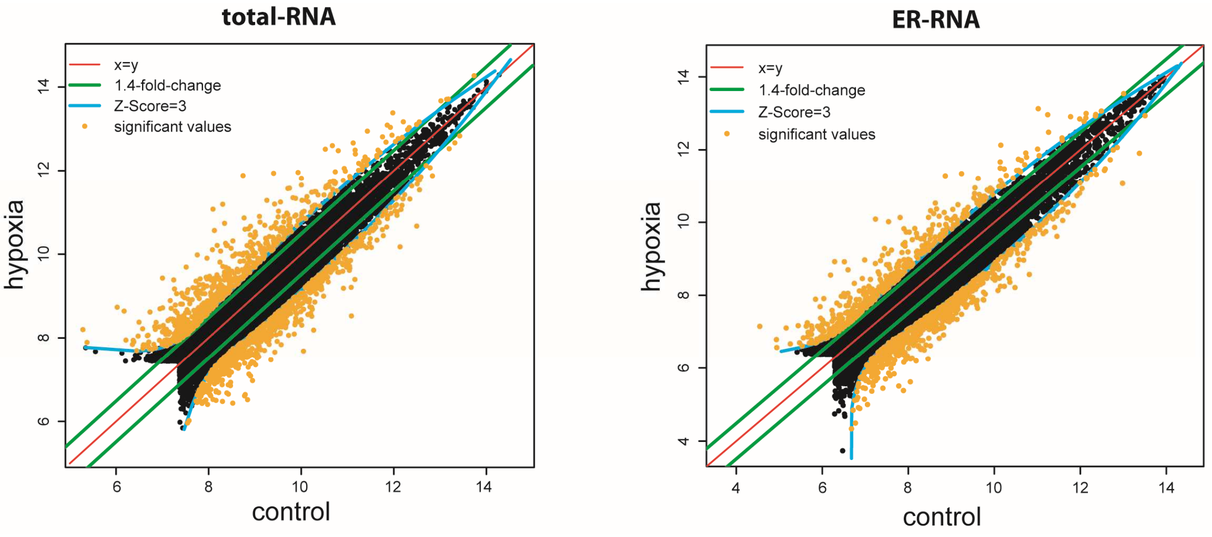

Figure S10: Model for selection of significantly changed candidates. Microarray data were evaluated for candidates significantly changed in hypoxia compared to control conditions. Levels were considered to be significant (yellow dots) if the fold change was >1.4 or <-1.4 (green line) and the z-score was >3 or <-3 (blue line). Significant data were calculated for total-RNA representing the expression level and ER-RNA indicating mRNA localization.

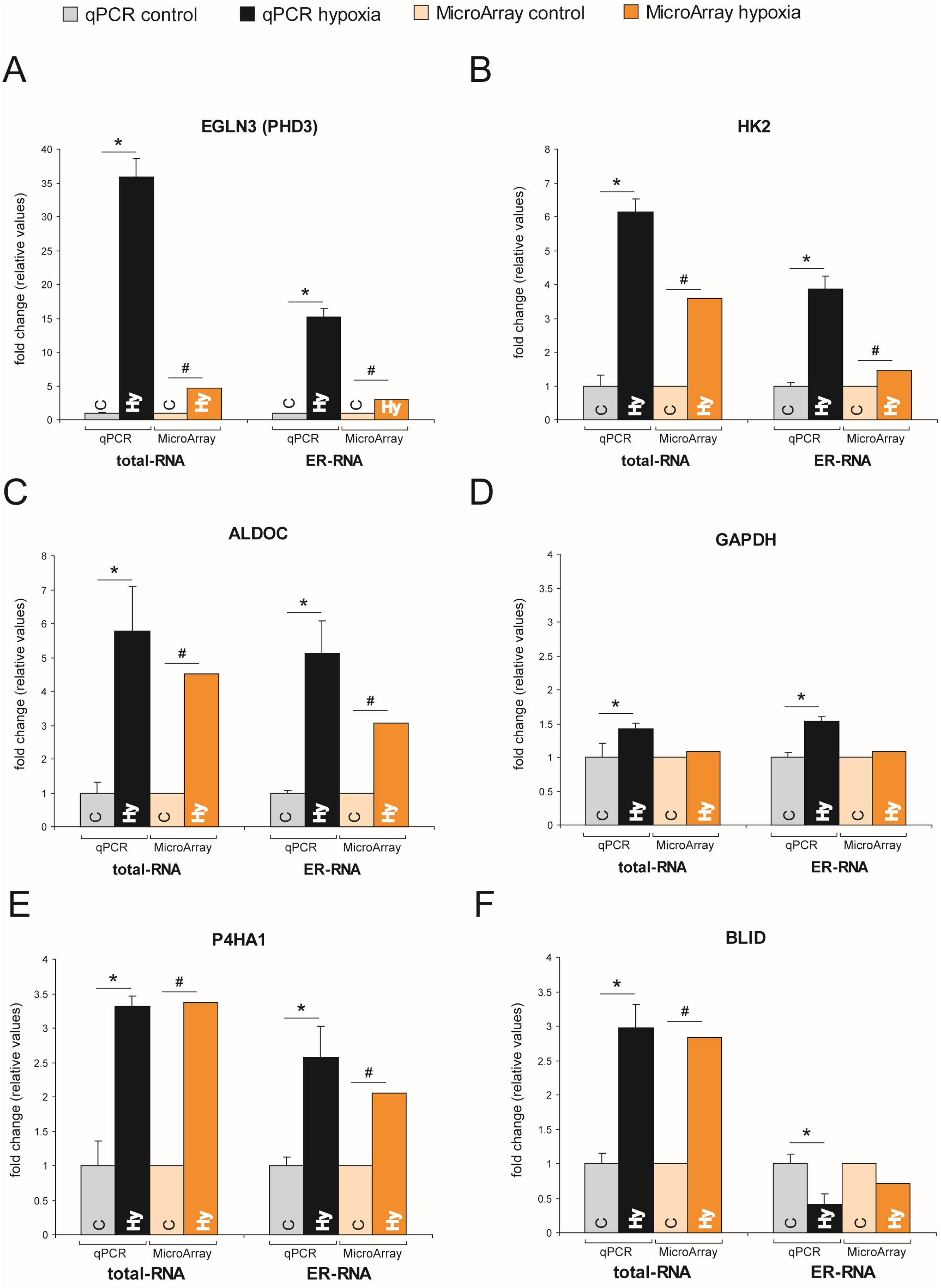

Figure S11: Verification of microarray data by qPCR. Microarray data of expression levels (total-RNA) and RNA-localization at the ER (ER-RNA) were verified by qPCR for selected candidates as indicated. Although the fold-change obtained by qPCR was higher in most cases, we found a good overlap in the decision of significantly changed candidates. An asterisk indicates significance with $p<0.05$ ($n=6$). A hash sign indicates significance according to the criteria: i) fold-change >1.4 or <-1.4 and ii) z-score >3 or <-3.

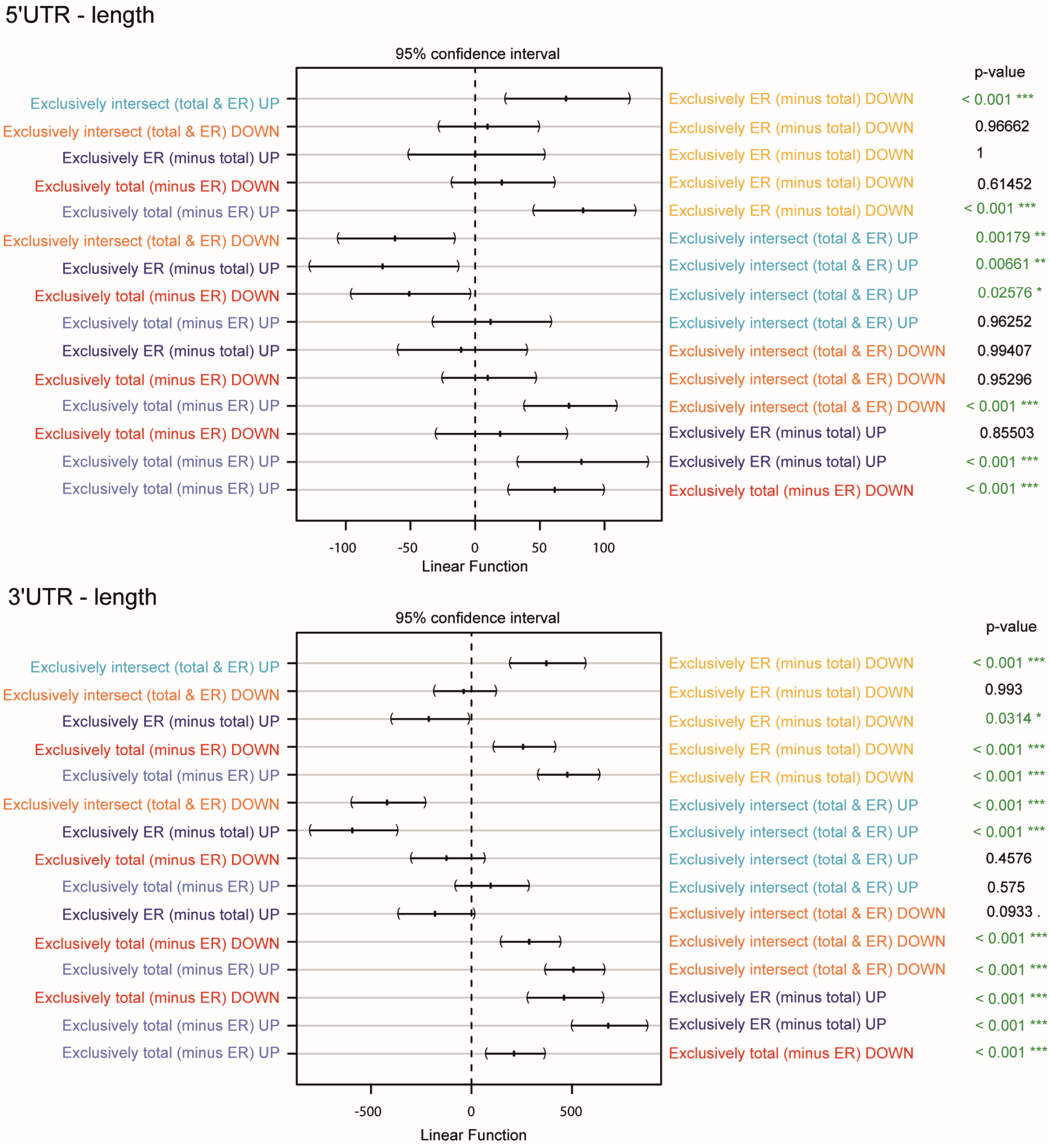

Figure S12: Comparison of group specific UTR length. 5'-UTR (upper panel) or 3'-UTR (lower panel) length of transcripts belonging to sub-groups that were regulated in hypoxia either by their expression and/or ER localization as described in Figure 4 were calculated. The statistics is based on a linear ANOVA model. *Post hoc* analysis was performed with the "Tukey Contrasts - Multiple Comparisons of Means" and the adjusted p-values are reported for the *post hoc* multiple comparisons.

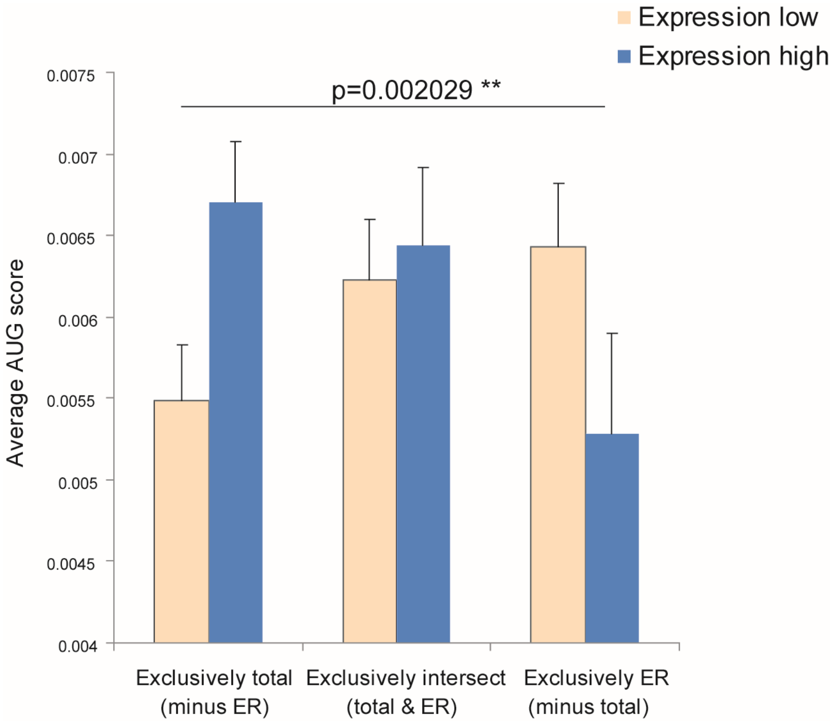

Figure S13: Gene centered mean AUG-score for down-regulated (Expression low) and up-regulated (Expression high) genes as described in Figure 4 with respect to cellular location. The AUG score describes the number AUG relative to transcript number, divided by the length of the UTR. Two factor ANOVA analysis reveals a statistically significant interaction between location and expression level for that score, suggesting that the mechanism(s) that lead to an AUG-dependent expression level differ in the analyzed cellular locations.

5'UTR - conservation

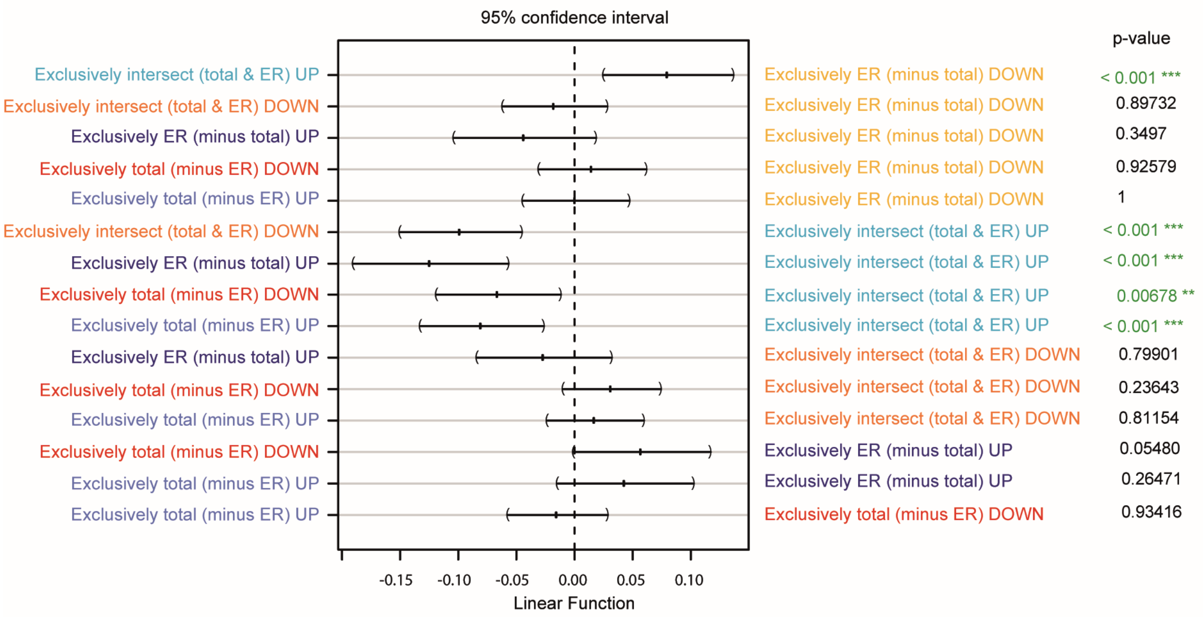

3'UTR - conservation

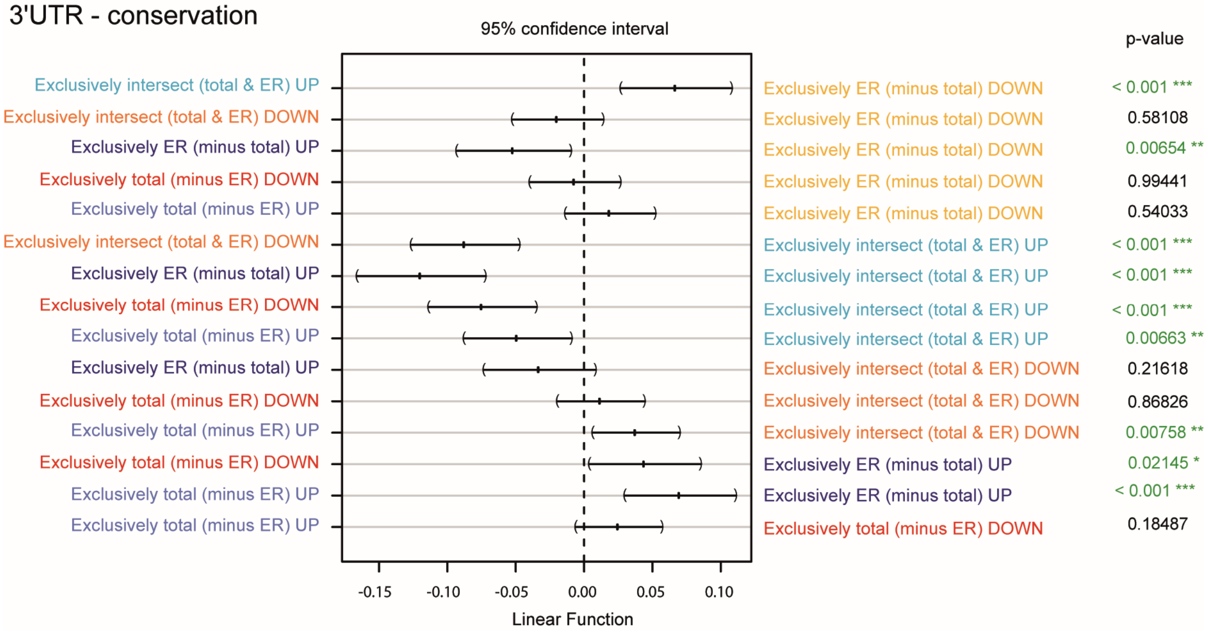

Figure S14: Comparison of group specific UTR conservation. 5'UTR (upper panel) or 3'UTR (lower panel) conservation scores of transcripts belonging to sub-groups that were regulated in hypoxia either by their expression and/or ER localization as described in Figure 4 were calculated. For calculation of the average evolutionary conservation score we obtained the base wise conservation score for the complete human genome from USCS (http://genome.ucsc.edu/). Based on this base wise mapping we calculated the average conservation score for each UTR of each transcript. The statistics is based on a linear ANOVA model. *Post hoc* analysis was performed with the "Tukey Contrasts - Multiple Comparisons of Means" and the adjusted p-values are reported for the *post hoc* multiple comparisons.

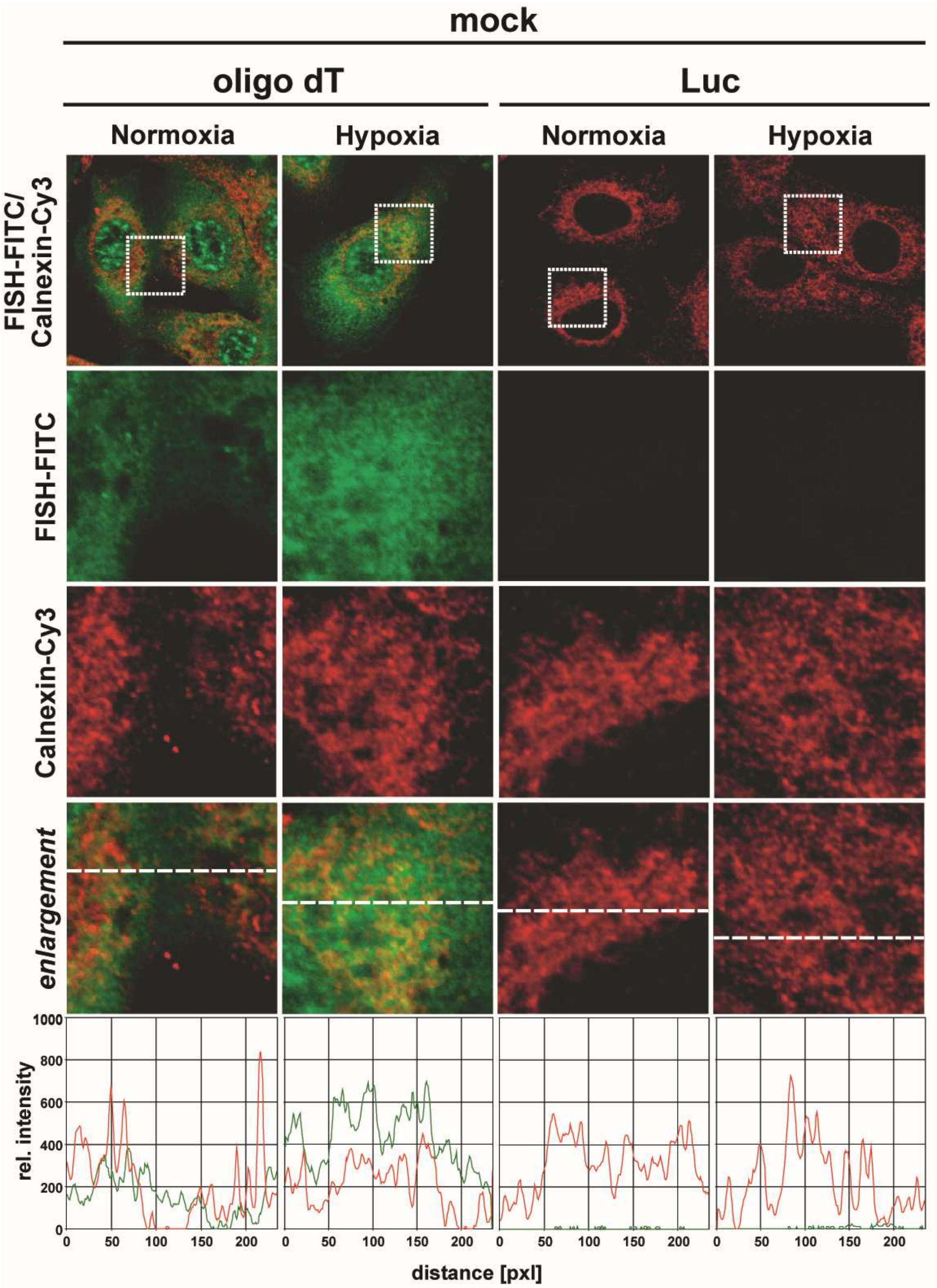

Figure S15: Dual fluorescence detection (IF-FISH) of RNA co-localization with the ER marker Calnexin in mock-transfected HT1080 cells. HT1080 cells were treated and analyzed as described in Figure 6 under normoxic or hypoxic (1% oxygen) conditions for 36 h and hybridized with oligo(dT) or Luciferase (Luc) mRNA probes (FITC, green) for *in situ* hybridization. Mock transfection resulted in no detectable Luciferase mRNA. Immunostaining of endogenous Calnexin (Cy3, red) was carried out using specific antibodies. Dotted squares indicate the magnified area represented in the enlargement. Cy3 and FITC fluorescence monitored along the dashed lines is shown as relative signal intensity in the bottom panel. Shown are control experiments related to Figure 6.

Table S1: Fold change in gene expression (total-RNA) and ER-localization (ER-RNA) of HIF-1 target genes. Micro-array data for HIF-1 target genes that were significantly regulated in HT1080 cells following 36 h of hypoxia are shown. Significant changes according to the fold-change and z-score are indicated in bold.

Gene symbol, name	fold change	
	total-RNA	ER-RNA
ALDOC, aldolase C	**3.99**	**3.93**
BNIP3L, BCL2/adenovirus E1B interacting protein 3-like	**1.78**	**1.82**
BNIP3, BCL2/adenovirus E1B 19kDa interacting protein 3	**3.81**	**3.29**
CA9, carbonic anhydrase IX	**8.82**	**5.00**
CITED2, Cbp/p300-interacting transactivator, 2	**1.86**	**2.11**
CCNG2, cyclin G2	**2.28**	**2.05**
ETS1, v-ets erythroblastosis virus E26 oncogene homolog 1	**1.77**	1.34
ENO2, enolase 2	**5.80**	**3.28**
GPI (AMF), glucose phosphate isomerase	**2.08**	**1.83**
GLUT1, glucose transporter 1	**1.53**	**1.64**
HK2, hexokinase 2	**3.63**	**1.86**
LRP1, low density lipoprotein-related protein 1	**2.30**	**1.56**
MMP9, matrix metalloproteinase 9	**1.80**	**1.52**
PFKL, phosphofructokinase L	**1.92**	**1.90**
PGK 1, phosphoglycerate kinase 1	**1.95**	**1.67**
P4HA1, prolyl 4-hydroxylase, alpha polypeptide I	**3.56**	**2.29**
TGF-alpha, transforming growth factor-alpha	**1.56**	1.37
VEGFC, vascular endothelial growth factor C	**2.06**	**1.53**
VIM, vimentin.	**1.53**	**1.49**

Table S2: GO enrichment analysis of transcript group "*Down regulated* - Exclusively total (minus ER)" as shown in Figure 4. The number of genes used for GO enrichment analysis is given in the table. Probe IDs from the Affymetrix HuGene 1.0 st v1 chip were used for WEB-based GEne SeT AnaLysis Toolkit (WEBGESTALT) gene ontology analysis (http://bioinfo.vanderbilt.edu/webgestalt/). (C) Number of reference genes in the category. (O) Number of genes in the gene set and also in the category. (E) Expected number in the category. (R) Ratio of enrichment. (rawP) p value from hypergeometric test. (adjP) p value adjusted by the multiple test adjustment. ***Please note:*** This group contained 4 non-coding RNAs (ncRNAs) that were not involved in the GO analysis.

Number of genes: **367**	**GO Analysis**	
	**Gene Ontology**	**Statistics**
biological process	cellular component organization----GO:0016043	C=2477;O=76;E=50.87;R=1.49; rawP=0.0001;adjP=0.0174
	cellular component biogenesis----GO:0044085	C=997;O=41;E=20.47;R=2.00; rawP=1.45e-05;adjP=0.0174
	tRNA processing----GO:0008033	C=72;O=8;E=1.48;R=5.41; rawP=0.0001;adjP=0.0174
	cellular response to stress----GO:0033554	C=536;O=26;E=11.01;R=2.36; rawP=4.38e-05;adjP=0.0174
	positive regulation of defense response to virus by host----GO:0002230	C=4;O=3;E=0.08;R=36.52; rawP=3.37e-05;adjP=0.0174
	ribonucleoprotein complex biogenesis----GO:0022613	C=175;O=13;E=3.59;R=3.62; rawP=6.61e-05;adjP=0.0174
	M phase----GO:0000279	C=352;O=19;E=7.23;R=2.63; rawP=0.0001;adjP=0.0174
	RNA processing----GO:0006396	C=525;O=24;E=10.78;R=2.23; rawP=0.0002;adjP=0.0243
	cellular response to stimulus----GO:0051716	C=785;O=32;E=16.12;R=1.99; rawP=0.0002;adjP=0.0243
	organelle fission----GO:0048285	C=253;O=15;E=5.20;R=2.89; rawP=0.0002;adjP=0.0243
molecular function	nuclease activity----GO:0004518	C=139;O=11;E=2.80;R=3.92; rawP=0.0001;adjP=0.0279
cellular component	organelle part----GO:0044422	C=3972;O=131;E=80.27;R=1.63; rawP=1.56e-10;adjP=2.93e-08
	intracellular organelle part----GO:0044446	C=3946;O=129;E=79.74;R=1.62; rawP=4.56e-10;adjP=4.29e-08
	organelle lumen----GO:0043233	C=1732;O=63;E=35.00;R=1.80; rawP=2.21e-06;adjP=8.31e-05
	membrane-enclosed lumen----GO:0031974	C=1767;O=64;E=35.71;R=1.79; rawP=2.08e-06;adjP=8.31e-05
	intracellular organelle lumen----GO:0070013	C=1691;O=62;E=34.17;R=1.81; rawP=2.09e-06;adjP=8.31e-05
	nuclear part----GO:0044428	C=1716;O=61;E=34.68;R=1.76; rawP=6.98e-06;adjP=0.0002

	macromolecular complex----GO:0032991	C=2887;O=88;E=58.34;R=1.51; rawP=2.19e-05;adjP=0.0006
	mitochondrial part----GO:0044429	C=544;O=26;E=10.99;R=2.37; rawP=4.49e-05;adjP=0.0011
	ribonucleoprotein complex----GO:0030529	C=466;O=23;E=9.42;R=2.44; rawP=7.69e-05;adjP=0.0016
	nuclear lumen----GO:0031981	C=1377;O=48;E=27.83;R=1.72; rawP=0.0001;adjP=0.0017

Supplementary Table S3: GO enrichment analysis of transcript group "*Down regulated* - Exclusively intersect (total & ER)" as shown in Figure 4. The number of genes used for GO enrichment analysis is given in the table. Probe IDs from the Affymetrix HuGene 1.0 st v1 chip were used for WEB-based GEne SeT AnaLysis Toolkit (WEBGESTALT) gene ontology analysis (http://bioinfo.vanderbilt.edu/webgestalt/). (C) Number of reference genes in the category. (O) Number of genes in the gene set and also in the category. (E) Expected number in the category. (R) Ratio of enrichment. (rawP) p value from hypergeometric test. (adjP) p value adjusted by the multiple test adjustment.

number of genes: **370**	**GO Analysis**	
	**Gene Ontology**	**Statistics**
biological process	cell cycle----GO:0007049	C=879;O=65;E=19.88;R=3.27; rawP=6.74e-18;adjP=2.12e-15
	mitosis----GO:0007067	C=246;O=30;E=5.56;R=5.39; rawP=4.20e-14;adjP=7.92e-12
	nuclear division----GO:0000280	C=246;O=30;E=5.56;R=5.39; rawP=4.20e-14;adjP=7.92e-12
	DNA packaging----GO:0006323	C=109;O=14;E=2.46;R=5.68; rawP=1.58e-07;adjP=4.66e-06
	DNA conformation change----GO:0071103	C=125;O=14;E=2.83;R=4.95; rawP=8.71e-07;adjP=2.05e-05
	cell cycle phase----GO:0022403	C=442;O=39;E=9.99;R=3.90; rawP=2.53e-13;adjP=2.39e-11
	M phase of mitotic cell cycle----GO:0000087	C=254;O=30;E=5.74;R=5.22; rawP=9.92e-14;adjP=1.34e-11
	organelle organization----GO:0006996	C=1323;O=60;E=29.91;R=2.01; rawP=1.05e-07;adjP=3.30e-06
	organelle fission----GO:0048285	C=253;O=30;E=5.72;R=5.24; rawP=8.93e-14;adjP=1.34e-11
	ribonucleoprotein complex biogenesis----GO:0022613	C=175;O=17;E=3.96;R=4.30; rawP=4.60e-07;adjP=1.20e-05
	RNA processing----GO:0006396	C=525;O=36;E=11.87;R=3.03; rawP=2.72e-09;adjP=9.87e-08
	ncRNA processing----GO:0034470	C=179;O=22;E=4.05;R=5.44; rawP=1.01e-10;adjP=5.29e-09
	tRNA metabolic process----GO:0006399	C=114;O=14;E=2.58;R=5.43; rawP=2.78e-07;adjP=7.94e-06
	rRNA processing----GO:0006364	C=89;O=12;E=2.01;R=5.96; rawP=7.21e-07;adjP=1.79e-05
	proteasomal protein catabolic process----GO:0010498	C=106;O=16;E=2.40;R=6.68; rawP=1.86e-09;adjP=7.63e-08
	regulation of ubiquitin-protein ligase activity during mitotic cell cycle----GO:0051439	C=67;O=16;E=1.51;R=10.56; rawP=1.25e-12;adjP=1.07e-10

	anaphase-promoting complex-dependent proteasomal ubiquitin-dependent protein catabolic process----GO:0031145	C=61;O=15;E=1.38;R=10.88; rawP=4.05e-12;adjP=3.18e-10
	positive regulation of ubiquitin-protein ligase activity during mitotic cell cycle----GO:0051437	C=64;O=15;E=1.45;R=10.37; rawP=8.62e-12;adjP=5.81e-10
	negative regulation of ubiquitin-protein ligase activity during mitotic cell cycle----GO:0051436	C=61;O=13;E=1.38;R=9.43; rawP=7.76e-10;adjP=3.85e-08
	cellular nitrogen compound metabolic process----GO:0034641	C=413;O=29;E=9.34;R=3.11; rawP=6.34e-08;adjP=2.06e-06
molecular function	catalytic activity----GO:0003824	C=4825;O=179;E=105.37;R=1.70; rawP=6.52e-18;adjP=1.88e-15
	ATP binding----GO:0005524	C=1404;O=62;E=30.66;R=2.02; rawP=4.90e-08;adjP=1.77e-06
	nucleotide binding----GO:0000166	C=2110;O=87;E=46.08;R=1.89; rawP=1.18e-09;adjP=1.71e-07
	nucleoside binding----GO:0001882	C=1532;O=68;E=33.46;R=2.03; rawP=7.45e-09;adjP=3.59e-07
	ribonucleotide binding----GO:0032553	C=1734;O=70;E=37.87;R=1.85; rawP=1.93e-07;adjP=5.07e-06
	threonine-type endopeptidase activity----GO:0004298	C=19;O=6;E=0.41;R=14.46; rawP=2.21e-06;adjP=4.91e-05
	exoribonuclease activity----GO:0004532	C=13;O=5;E=0.28;R=17.61; rawP=5.36e-06;adjP=0.0001
	coenzyme binding----GO:0050662	C=170;O=14;E=3.71;R=3.77; rawP=2.19e-05;adjP=0.0004
	hydrolase activity----GO:0016787	C=2125;O=73;E=46.41;R=1.57; rawP=3.82e-05;adjP=0.0006
	3'-5'-exoribonuclease activity----GO:0000175	C=10;O=4;E=0.22;R=18.32; rawP=4.22e-05;adjP=0.0006
	ligase activity, forming carbon-oxygen bonds----GO:0016875	C=45;O=7;E=0.98;R=7.12; rawP=4.93e-05;adjP=0.0006
	aminoacyl-tRNA ligase activity----GO:0004812	C=45;O=7;E=0.98;R=7.12; rawP=4.93e-05;adjP=0.0006
	aspartate-tRNA ligase activity----GO:0004815	C=4;O=3;E=0.09;R=34.34; rawP=4.06e-05;adjP=0.0006
	asparagine-tRNA ligase activity----GO:0004816	C=2;O=2;E=0.04;R=45.79; rawP=0.0005;adjP=0.0044
	lyase activity----GO:0016829	C=144;O=12;E=3.14;R=3.82; rawP=7.54e-05;adjP=0.0009
	cyclohydrolase activity----GO:0019238	C=5;O=3;E=0.11;R=27.47; rawP=9.99e-05;adjP=0.0012
	oxidoreductase activity----GO:0016491	C=642;O=29;E=14.02;R=2.07; rawP=0.0002;adjP=0.0021

	transferase activity----GO:0016740	C=1591;O=55;E=34.75;R=1.58; rawP=0.0004;adjP=0.0039
	methylenetetrahydrofolate dehydrogenase (NADP+) activity----GO:0004488	C=2;O=2;E=0.04;R=45.79; rawP=0.0005;adjP=0.0044
	glycoprotein endo-alpha-1,2-mannosidase activity----GO:0004569	C=2;O=2;E=0.04;R=45.79; rawP=0.0005;adjP=0.0044
	RNA binding----GO:0003723	C=668;O=28;E=14.59;R=1.92; rawP=0.0007;adjP=0.0060
	pyridoxal phosphate binding----GO:0030170	C=53;O=6;E=1.16;R=5.18; rawP=0.0010;adjP=0.0074
	NADH dehydrogenase activity----GO:0003954	C=36;O=5;E=0.79;R=6.36; rawP=0.0010;adjP=0.0074
	vitamin B6 binding----GO:0070279	C=53;O=6;E=1.16;R=5.18; rawP=0.0010;adjP=0.0074
	isomerase activity----GO:0016853	C=120;O=9;E=2.62;R=3.43; rawP=0.0013;adjP=0.0088
cellular component	intracellular membrane-bounded organelle----GO:0043231	C=7534;O=227;E=156.26;R=1.45; rawP=1.41e-16;adjP=3.48e-15
	non-membrane-bounded organelle----GO:0043228	C=2426;O=86;E=50.32;R=1.71; rawP=1.69e-07;adjP=1.12e-06
	mitochondrion----GO:0005739	C=993;O=58;E=20.60;R=2.82; rawP=4.57e-13;adjP=6.76e-12
	macromolecular complex----GO:0032991	C=2887;O=120;E=59.88;R=2.00; rawP=1.03e-15;adjP=2.04e-14
	nucleus----GO:0005634	C=4780;O=151;E=99.14;R=1.52; rawP=4.93e-10;adjP=5.14e-09
	nuclear part----GO:0044428	C=1716;O=81;E=35.59;R=2.28; rawP=4.78e-13;adjP=6.76e-12
	chromosomal part----GO:0044427	C=358;O=29;E=7.43;R=3.91; rawP=3.81e-10;adjP=4.44e-09
	nuclear lumen----GO:0031981	C=1377;O=64;E=28.56;R=2.24; rawP=4.92e-10;adjP=5.14e-09
	cytoplasm----GO:0005737	C=6930;O=204;E=143.73;R=1.42; rawP=3.09e-12;adjP=4.08e-11
	protein complex----GO:0043234	C=2370;O=96;E=49.16;R=1.95; rawP=1.52e-11;adjP=1.88e-10
	proteasome complex----GO:0000502	C=59;O=12;E=1.22;R=9.81; rawP=2.40e-09;adjP=2.26e-08
	envelope----GO:0031975	C=557;O=33;E=11.55;R=2.86; rawP=5.89e-08;adjP=4.17e-07
	microtubule cytoskeleton----GO:0015630	C=514;O=29;E=10.66;R=2.72; rawP=1.07e-06;adjP=5.89e-06

Supplementary Table S4: GO enrichment analysis of transcript group "*Down regulated* - Exclusively ER (minus total)" as shown in Figure 4. The number of genes used for GO enrichment analysis is given in the table. Probe IDs from the Affymetrix HuGene 1.0 st v1 chip were used for WEB-based GEne SeT AnaLysis Toolkit (WEBGESTALT) gene ontology analysis (http://bioinfo.vanderbilt.edu/webgestalt/). (C) Number of reference genes in the category. (O) Number of genes in the gene set and also in the category. (E) Expected number in the category. (R) Ratio of enrichment. (rawP) p value from hypergeometric test. (adjP) p value adjusted by the multiple test adjustment. ***Please note:*** This group contained 1 non-coding RNA (ncRNA) that was not involved in the GO analysis.

number of genes: **318**	**GO Analysis**	
	**Gene Ontology**	**Statistics**
biological process	RNA processing----GO:0006396	C=525;O=29;E=8.80;R=3.30; rawP=1.52e-08;adjP=1.08e-05
	mitotic cell cycle----GO:0000278	C=446;O=24;E=7.47;R=3.21; rawP=4.71e-07;adjP=0.0001
	cell cycle process----GO:0022402	C=586;O=28;E=9.82;R=2.85;rawP=5.57e-07;adjP=0.0001
	cellular component biogenesis----GO:0044085	C=997;O=38;E=16.70;R=2.27; rawP=1.40e-06;adjP=0.0002
	ncRNA processing----GO:0034470	C=179;O=14;E=3.00;R=4.67;rawP=1.89e-06;adjP=0.0002
	cell cycle----GO:0007049	C=879;O=35;E=14.73;R=2.38; rawP=1.45e-06;adjP=0.0002
	cell division----GO:0051301	C=313;O=18;E=5.24;R=3.43; rawP=5.50e-06;adjP=0.0005
	chromosome segregation----GO:0007059	C=80;O=9;E=1.34;R=6.71;rawP=7.33e-06;adjP=0.0005
	DNA metabolic process----GO:0006259	C=523;O=24;E=8.76;R=2.74; rawP=7.58e-06;adjP=0.0005
	cellular metabolic process----GO:0044237	C=6973;O=150;E=116.83;R=1.28; rawP=4.72e-06;adjP=0.0005
molecular function	methyltransferase activity----GO:0008168	C=162;O=15;E=2.70;R=5.55; rawP=8.67e-08;adjP=7.07e-06
	transferase activity, transferring one-carbon groups----GO:0016741	C=165;O=15;E=2.75;R=5.45; rawP=1.11e-07;adjP=7.07e-06
	catalytic activity----GO:0003824	C=4825;O=120;E=80.54;R=1.49; rawP=8.60e-08;adjP=7.07e-06
	RNA binding----GO:0003723	C=668;O=29;E=11.15;R=2.60; rawP=2.34e-06;adjP=0.0001
	hydrolase activity, acting on acid anhydrides----GO:0016817	C=724;O=29;E=12.09;R=2.40; rawP=1.11e-05;adjP=0.0003
	hydrolase activity, acting on acid anhydrides, in phosphorus-containing anhydrides----GO:0016818	C=719;O=29;E=12.00;R=2.42; rawP=9.75e-06;adjP=0.0003

	pyrophosphatase activity----GO:0016462	C=716;O=29;E=11.95;R=2.43; rawP=9.00e-06;adjP=0.0003
	nucleotide binding----GO:0000166	C=2110;O=60;E=35.22;R=1.70; rawP=1.58e-05;adjP=0.0004
	nucleoside-triphosphatase activity----GO:0017111	C=688;O=26;E=11.48;R=2.26; rawP=8.59e-05;adjP=0.0017
	single-stranded DNA binding----GO:0006330	C=6;O=3;E=0.10;R=29.95; rawP=8.85e-05;adjP=0.0017
cellular component	intracellular organelle lumen----GO:0070013	C=1691;O=75;E=26.64;R=2.82; rawP=1.60e-17;adjP=1.37e-15
	membrane-enclosed lumen----GO:0031974	C=1767;O=77;E=27.84;R=2.77; rawP=1.36e-17;adjP=1.37e-15
	intracellular----GO:0005622	C=10361;O=217;E=163.24;R=1.33; rawP=2.36e-17;adjP=1.37e-15
	organelle lumen----GO:0043233	C=1732;O=75;E=27.29;R=2.75; rawP=6.14e-17;adjP=2.67e-15
	intracellular part----GO:0044424	C=10034;O=212;E=158.08;R=1.34; rawP=2.04e-16;adjP=7.10e-15
	intracellular membrane-bounded organelle----GO:0043231	C=7534;O=178;E=118.70;R=1.50; rawP=1.76e-15;adjP=4.82e-14
	membrane-bounded organelle----GO:0043227	C=7540;O=178;E=118.79;R=1.50; rawP=1.94e-15;adjP=4.82e-14
	intracellular organelle part----GO:0044446	C=3946;O=118;E=62.17;R=1.90; rawP=5.10e-15;adjP=1.11e-13
	organelle part----GO:0044422	C=3972;O=118;E=62.58;R=1.89; rawP=8.50e-15;adjP=1.64e-13
	nuclear part----GO:0044428	C=1716;O=68;E=27.04;R=2.52; rawP=2.34e-13;adjP=4.07e-12

Supplementary Table S5: GO enrichment analysis of transcript group "*Up regulated* - Exclusively total (minus ER)" as shown in Figure 4. The number of genes used for GO enrichment analysis is given in the table. Probe IDs from the Affymetrix HuGene 1.0 st v1 chip were used for WEB-based GEne SeT AnaLysis Toolkit (WEBGESTALT) gene ontology analysis (http://bioinfo.vanderbilt.edu/webgestalt/). (C) Number of reference genes in the category. (O) Number of genes in the gene set and also in the category. (E) Expected number in the category. (R) Ratio of enrichment. (rawP) p value from hypergeometric test. (adjP) p value adjusted by the multiple test adjustment. ***Please note:*** This group contained 64 non-coding RNAs (ncRNAs) that were not involved in the GO analysis.

number of genes: **425**	**GO Analysis**	
	**Gene Ontology**	**Statistics**
biological process	cholesterol metabolic process----GO:0008203	C=92;O=7;E=1.36;R=5.16; rawP=0.0004;adjP=0.0384
	vasculature development----GO:0001944	C=271;O=13;E=4.00;R=3.25; rawP=0.0002;adjP=0.0384
	positive regulation of locomotion----GO:0040017	C=93;O=7;E=1.37;R=5.10; rawP=0.0004;adjP=0.0384
	pyramidal neuron differentiation----GO:0021859	C=2;O=2;E=0.03;R=67.78; rawP=0.0002;adjP=0.0384
	positive regulation of cellular component movement----GO:0051272	C=93;O=7;E=1.37;R=5.10; rawP=0.0004;adjP=0.0384
	sterol biosynthetic process----GO:0016126	C=42;O=5;E=0.62;R=8.07; rawP=0.0004;adjP=0.0384
	blood vessel development----GO:0001568	C=265;O=13;E=3.91;R=3.33; rawP=0.0002;adjP=0.0384
molecular function	transition metal ion binding----GO:0046914	C=2609;O=57;E=35.57;R=1.60; rawP=0.0001;adjP=0.0195
	zinc ion binding----GO:0008270	C=2163;O=49;E=29.49;R=1.66; rawP=0.0002;adjP=0.0195
cellular component	--	--

Supplementary Table S6: GO enrichment analysis of transcript group "*Up regulated* - Exclusively intersect (total & ER)" as shown in Figure 4. The number of genes used for GO enrichment analysis is given in the table. Probe IDs from the Affymetrix HuGene 1.0 st v1 chip were used for WEB-based GEne SeT AnaLysis Toolkit (WEBGESTALT) gene ontology analysis (http://bioinfo.vanderbilt.edu/webgestalt/). (C) Number of reference genes in the category. (O) Number of genes in the gene set and also in the category. (E) Expected number in the category. (R) Ratio of enrichment. (rawP) p value from hypergeometric test. (adjP) p value adjusted by the multiple test adjustment. ***Please note:*** This group contained 6 non-coding RNAs (ncRNAs) that were not involved in the GO analysis.

number of genes: **190**	**GO Analysis**	
	**Gene Ontology**	**Statistics**
biological process	response to hypoxia----GO:0001666	C=127;O=11;E=1.20;R=9.20; rawP=2.94e-08;adjP=1.17e-05
	response to oxygen levels----GO:0070482	C=133;O=11;E=1.25;R=8.78; rawP=4.75e-08;adjP=1.17e-05
	glycolysis----GO:0006096	C=51;O=6;E=0.48;R=12.50; rawP=7.88e-06;adjP=0.0013
	glucose catabolic process----GO:0006007	C=62;O=6;E=0.58;R=10.28; rawP=2.47e-05;adjP=0.0017
	hexose catabolic process----GO:0019320	C=72;O=6;E=0.68;R=8.85; rawP=5.82e-05;adjP=0.0036
	monosaccharide catabolic process----GO:0046365	C=74;O=6;E=0.70;R=8.61; rawP=6.79e-05;adjP=0.0037
	alcohol catabolic process----GO:0046164	C=84;O=6;E=0.79;R=7.59; rawP=0.0001;adjP=0.0045
	cellular carbohydrate catabolic process----GO:0044275	C=92;O=6;E=0.87;R=6.93; rawP=0.0002;adjP=0.0082
	sequestering of actin monomers----GO:0042989	C=4;O=2;E=0.04;R=53.11; rawP=0.0005;adjP=0.0176
	oxidation reduction----GO:0055114	C=605;O=15;E=5.70;R=2.63; rawP=0.0006;adjP=0.0185
molecular function	L-ascorbic acid binding----GO:0031418	C=20;O=7;E=0.18;R=39.01; rawP=2.79e-10;adjP=4.16e-08
	dioxygenase activity----GO:0051213	C=65;O=8;E=0.58;R=13.72; rawP=1.11e-07;adjP=4.66e-06
	oxidoreductase activity----GO:0016701	C=66;O=8;E=0.59;R=13.51; rawP=1.25e-07;adjP=4.66e-06
	peptidyl-proline dioxygenase activity----GO:0031543	C=7;O=3;E=0.06;R=47.77; rawP=2.41e-05;adjP=0.0005
	vitamin binding----GO:0019842	C=128;O=8;E=1.15;R=6.97;rawP=1.96e-05;adjP=0.0005
	carboxylic acid binding----GO:0031406	C=140;O=8;E=1.26;R=6.37; rawP=3.74e-05;adjP=0.0007
	cadmium ion binding----GO:0046870	C=10;O=3;E=0.09;R=33.44; rawP=8.09e-05;adjP=0.0013

	copper ion binding----GO:0005507	C=65;O=5;E=0.58;R=8.57; rawP=0.0003;adjP=0.0034
	procollagen-lysine 5-dioxygenase activity----GO:0008475	C=3;O=2;E=0.03;R=74.30; rawP=0.0002;adjP=0.0027
	monosaccharide binding----GO:0048029	C=39;O=3;E=0.35;R=8.57; rawP=0.0051;adjP=0.0304
cellular component	--	--

Supplementary Table S7: GO enrichment analysis of transcript group "*Down regulated* - Exclusively ER (minus total)" as shown in Figure 4. The number of genes used for GO enrichment analysis is given in the table. Probe IDs from the Affymetrix HuGene 1.0 st v1 chip were used for WEB-based GEne SeT AnaLysis Toolkit (WEBGESTALT) gene ontology analysis (http://bioinfo.vanderbilt.edu/webgestalt/). (C) Number of reference genes in the category. (O) Number of genes in the gene set and also in the category. (E) Expected number in the category. (R) Ratio of enrichment. (rawP) p value from hypergeometric test. (adjP) p value adjusted by the multiple test adjustment. ***Please note:*** This group contained 113 non-coding RNAs (ncRNAs) that were not involved in the GO analysis.

number of genes: **296**	**GO Analysis**	
	**Gene Ontology**	**Statistics**
biological process	translational elongation----GO:0006414	C=97;O=4;E=0.59;R=6.78; rawP=0.0029;adjP=0.1804
molecular function	cadmium ion binding----GO:0046870	C=10;O=2;E=0.06;R=34.23; rawP=0.0015;adjP=0.0540
	intramolecular oxidoreductase activity----GO:0016860	C=38;O=3;E=0.22;R=13.51;rawP=0.0014;adjP=0.0540
	copper ion binding----GO:0005507	C=65;O=3;E=0.38;R=7.90;rawP=0.0065;adjP=0.1404
	protein binding----GO:0005515	C=7713;O=54;E=45.06;R=1.20;rawP=0.0312;adjP=0.4296
cellular component	--	--

Supplemental Experimental Procedures

RNA quantification by real-time PCR (qPCR)

The following primers were used for the amplification reactions:

P4HB forward (fw): AGA CTC ACA TCC TGC TGT TC,

P4HB reverse (re): TAC TTG GTC ATC TCC TCC TC;

P4HA1(fw): CCA CAG CAG AGG AAT TAC AG,

P4HA1(re): ACA CTA GCT CCA ACT TCA GG;

β-Actin(fw): TGA AGT GTG ACG TGG ACA TC,

β-Actin(re): GTC ATA GTC CGC CTA GAA GC;

HIF-1α (fw): CCA ACA GTA ACC AAC CTC AG,

HIF-1α (re): TCC TGT GGT GAC TTG TCC TT;

BLID(fw): GCC ACT GGA GGC GCT CTT GGG T,

BLID(re): TTC CGC TTC ATG GCA GAG GAG CCA;

HK2(fw): CAC CCT CGC CGG TAG CCT TCT TT,

HK2(re): GGG TGA GTG GTG GCT CCA AGC CC;

18S rRNA(fw): GAT CAA AAC CAA CCC GGT CA,

18S rRNA(re): CCG TTT CTC AGG CTC CCT CT,
